# Identification of Novel Small Molecule Inhibitors of Oncogenic RET Kinase

**DOI:** 10.1371/journal.pone.0128364

**Published:** 2015-06-05

**Authors:** Marialuisa Moccia, Qingsong Liu, Teresa Guida, Giorgia Federico, Annalisa Brescia, Zheng Zhao, Hwan Geun Choi, Xianming Deng, Li Tan, Jinhua Wang, Marc Billaud, Nathanael S. Gray, Francesca Carlomagno, Massimo Santoro

**Affiliations:** 1 Dipartimento di Medicina Molecolare e Biotecnologie Mediche, Università di Napoli “Federico II”, Naples, Italy; 2 Istituto di Endocrinologia ed Oncologia Sperimentale del CNR, Naples, Italy; 3 Department of Cancer Biology, Dana Farber Cancer Institute, Boston, Massachusetts United States of America; 4 Department of Biological Chemistry and Molecular Pharmacology, Harvard Medical School, Boston, Massachusetts, United States of America; 5 High Magnetic Field Laboratory, Chinese Academy of Sciences, Hefei, Anhui, P.R. China; 6 Institut Albert Bonniot, CRI INSERM/UJF U823, La Tronche Cedex, France; IPATIMUP/Faculty of Medicine of the University of Porto, PORTUGAL

## Abstract

Oncogenic mutation of the *RET* receptor tyrosine kinase is observed in several human malignancies. Here, we describe three novel type II RET tyrosine kinase inhibitors (TKI), ALW-II-41-27, XMD15-44 and HG-6-63-01, that inhibit the cellular activity of oncogenic *RET* mutants at two digit nanomolar concentration. These three compounds shared a 3-trifluoromethyl-4-methylpiperazinephenyl pharmacophore that stabilizes the ‘DFG-out’ inactive conformation of RET activation loop. They blocked RET-mediated signaling and proliferation with an IC50 in the nM range in fibroblasts transformed by the *RET*/C634R and *RET*/M918T oncogenes. They also inhibited autophosphorylation of several additional oncogenic *RET-*derived point mutants and chimeric oncogenes. At a concentration of 10 nM, ALW-II-41-27, XMD15-44 and HG-6-63-01 inhibited RET kinase and signaling in human thyroid cancer cell lines carrying oncogenic *RET* alleles; they also inhibited proliferation of cancer, but not non-tumoral Nthy-ori-3-1, thyroid cells, with an IC50 in the nM range. The three compounds were capable of inhibiting the ‘gatekeeper’ V804M mutant which confers substantial resistance to established RET inhibitors. In conclusion, we have identified a type II TKI scaffold, shared by ALW-II-41-27, XMD15-44 and HG-6-63-01, that may be used as novel lead for the development of novel agents for the treatment of cancers harboring oncogenic activation of RET.

## Introduction

The *REarranged during Transfection* (*RET*) gene codes for a single pass transmembrane tyrosine kinase (TK) receptor that is mutated in several human cancers [1]. In approximately 20% of human papillary thyroid carcinoma (PTC), *RET* exons encoding the tyrosine kinase domain are fused to the promoter region and the 5’-ter exons of heterologous genes, generating chimeric oncogenes, such as *CCDC6-RET* (*RET/PTC1*) or *NCOA4-RET* (*RET/PTC3*) [1, 2]. Missense germline and somatic point mutations of *RET* are associated to familial (95%) and sporadic (50%) cases of medullary thyroid carcinoma (MTC), respectively. MTC associated *RET* mutations commonly target cysteine residues in the extracellular domain or the intracellular tyrosine kinase domain [1–3].

In roughly 1% of non small cell lung cancers (NSCLC), particularly in adenocarcinoma, chromosomal inversions cause the fusion of the *RET*-encoded TK domain to different exons of *KIF5B* (kinesin family member 5B) gene or, less commonly, to *CCDC6*, *NCOA4* or TRIMM33 [4–8]. Finally, in patients affected by myeloproliferative disorders (MPD), such as chronic myelomonocytic leukemia or primary myelofibrosis, oncogenic *RET* fusions with *BCR* or *FGFR1OP* genes were identified [9, 10].

PTC-, NSCLC- and MPD-associated *RET* rearrangements and MTC-associated *RET* point mutations induce an oncogenic conversion of RET gene product by promoting ligand-independent kinase activation [1, 11]. Unscheduled RET TK activation results in its constitutive autophosphorylation on specific tyrosine residues, such as Y905 and Y1062, in the intracellular domain. This, in turn, switches-on several signalling pathways, like the SHC/RAS/MAPK pathway, that support cell transformation [1, 11].

Based on this knowledge, RET targeting in cancer has been exploited *via* the identification of small molecule RET tyrosine kinase inhibitors (TKI) [12, 13]. Two of them, vandetanib (ZD6474) and cabozantinib (XL184), have been approved for locally advanced or metastatic MTC [14, 15]. Vandetanib binds to the active conformation of RET kinase (DFG-in) in the ATP-binding pocket and it is therefore a type I kinase inhibitor [16, 17]. Though *in vivo* molecular mechanisms of acquired resistance are still unknown, RET mutations V804M/L or Y806C are able to cause a 50- (V804M/L) and 10-fold (Y806C) increase of *in vitro* IC_50_ dose of vandetanib for RET [18, 19] and V804M causes resistance to cabozantinib, as well [20]. It is still unknown whether such mutations might be involved in building resistance in patients.

Here we describe the identification and characterization of ALW-II-41-27 [21, 22], XMD15-44 [23] and HG-6-63-01 as novel potent inhibitors of RET kinase. These compounds were type II inhibitors, designed to bind to the ‘DFG-out’ inactive kinase conformation, and all contain a 3-trifluoromethyl-4-methylpiperazinephenyl pharmacophore which occupies the hydrophobic pocket created by the rearrangement of the activation loop [23]. Thus, the common structure shared by the three compounds may represent a novel scaffold to generate potent and selective type II TKIs for cancers that exhibit constitutively active *RET* signaling.

## Materials and Methods

### Compounds

Compounds were synthesized in the Gray’s laboratory according to published procedures [21, 23], dissolved in dimethyl sulfoxide (DMSO) at 10 mM concentration and stored at -80°C. The synthetic procedure and characterization for HG-6-63-01 is provided in the Supplemental informations (S1 Methods). Final dosing solution was prepared on the day of use by dilution of the stock solution in cell growth media.

### Molecule modeling

Though currently there are seven available X-ray structures of RET kinase in the public domain, all of them exhibit the ‘DFG-in’ active conformation of the activation loop and would not accommodate type II inhibitors. Therefore, here we first built the DFG-out model of RET kinase using the homology modelling method based on the RET sequence and the high-homology structure (PDB ID: 3DZQ) as the template with Swiss-model web server [24–27]. Then we used the autodock4.0 software to dock each ligand into the modeled DFG-out conformation of RET. The ligands were constructed by the online-tool: CORINA (http://www.molecular-networks.com). Lamarckian genetic algorithm with the default parameters was performed to get the candidate compounds. Then the docked compounds were clustered and sorted based on the binding free energy. The compound with the lowest binding free energy was shown as the binding mode.

### Cell cultures

Parental and RET-transformed mouse NIH3T3 cells (*RET/C634R* or *RET/M918T*) [28] were cultured in Dulbecco’s modified Eagle’s medium (DMEM) supplemented with 5% calf serum, 2 mM L-glutamine and 100 units/ml penicillin-streptomycin (GIBCO, Paisley, PA). Parental rat RAT1 fibroblasts and RAT1 cells transformed by *RET/C634R*, *RET/E768D*, *RET/L790F*, *RET/V804L*, *RET/V804M*, *RET/A883F*, *RET/S891A* and *RET/M918T* [29] were cultured in RPMI with 10% fetal calf serum, 2 mM L-glutamine and 100 units/ml penicillin-streptomycin (GIBCO). Human HEK 293 cells were from American Type Culture Collection (ATCC, Manassas, VA) and were grown in DMEM supplemented with 10% fetal calf serum, 2 mM L-glutamine, and 100 units/ml penicillin-streptomycin (GIBCO). *KIF5B/RET* cDNA (variant 2) was cloned in pBABE by fusing the 5’-terminal portion of *KIF5B* cDNA fragment (exons 1–16, encoding residues 1–638) to the 3’-terminal portion of RET cDNA (exons 12–20, encoding residues 713–1072, including the tyrosine kinase domain). Transient transfections of pBABE-*RET/PTC1*,*-RET/PTC3* and-*KIF5B/RET* vectors were carried-out with the lipofectamine reagent according to manufacturer's instructions (GIBCO). All the *RET* constructs used to transfect murine fibroblasts encoded the short isoform of the RET protein (RET-9). Ba/F3 cells stably expressing RET/PTC3 protein were generated by transfecting long isoform RET/PTC3 (RET-51) by electroporation. Parental murine Ba/F3 cells were from ATCC. Parental Ba/F3 and Ba/F3-RET/PTC3 cells were cultured in RPMI supplemented with 10% fetal calf serum, 2 mM L-glutamine and 100 units/ml penicillin-streptomycin (GIBCO). Parental Ba/F3 cells were grown in the presence of 10 ng/ml IL-3.

TT human cell line was obtained in 2002 from ATCC and authenticated by *RET* genotyping; it was derived from a MTC and harbors a cysteine 634 to tryptophan (C634W) *RET* mutation [30]. TT cells were grown in RPMI 1640 supplemented with 20% fetal calf serum (GIBCO). MZ-CRC-1 human cells were kindly provided in 2009 by Robert F. Gagel (MD Anderson, Houston, TX) and authenticated by *RET* genotyping. MZ-CRC-1 cells were derived from a malignant pleural effusion from a patient with metastatic MTC and were found to bear a heterozygous (ATG to ACG) transition in *RET* resulting in the MEN2B-associated substitution of threonine 918 for methionine (M918T) [31]. MZ-CRC-1 cells were grown in DMEM supplemented with 10% fetal calf serum (GIBCO). Nthy-ori 3–1, a human thyroid follicular epithelial cell line immortalized by the SV40 large T gene, was obtained from European Collection of Cell Cultures (ECACC) (Wiltshire, UK) in 2010. Nthy-ori 3–1 was grown in DMEM supplemented with 10% fetal calf serum (GIBCO). TPC-1 cells were obtained in 1990 from M. Nagao (National Cancer Center Research Institute, Tokyo, Japan) and authenticated by *RET/PTC* genotyping [32]; they were grown in DMEM supplemented with 10% fetal calf serum (GIBCO).

### Immunoblotting

Protein lysates were prepared according to standard procedures. Briefly, cells were lysed in a buffer containing 50 mM N-2-hydroxyethylpiperazine-N'-2-ethanesulfonic acid (HEPES; pH 7.5), 1% (vol/vol) Triton X-100, 150 mM NaCl, 5 mM EGTA, 50 mM NaF, 20 mM sodium pyrophosphate, 1 mM sodium vanadate, 2 mM phenylmethylsulphonyl fluoride (PMSF) and 1 μg/ml aprotinin. Lysates were clarified by centrifugation at 10,000 Xg for 15 min. Lysates containing comparable amounts of proteins, estimated by a modified Bradford assay (Bio-Rad, Munich, Germany), were subjected to direct Western blot. Immune complexes were detected with the enhanced chemiluminescence kit (Amersham Pharmacia Biotech, Little Chalfort, UK). Signal intensity was analyzed at the Phosphorimager (Typhoon 8600, Amersham Pharmacia Biotech) interfaced with the ImageQuant software. Anti-phospho-SHC (#07206), that recognizes SHC proteins when phosphorylated on Y317 was from Merck Millipore (Darmstadt, Germany). Anti-MAPK (#4695) and anti-phospho-MAPK (#4370), specific for p44/42MAPK (ERK1/2) phosphorylated on Thr202/Tyr204, anti-VEGFRII (#2479) and anti-phospho-VEGFRII (#2478), that recognizes VEGF Receptor 2 when phosphorylated on Y1175, were from Cell Signaling (Beverly, MA).

Anti-SHC (#H-108) was from Santa Cruz Biotechnology (Santa Cruz, CA). Anti-RET is a polyclonal antibody raised against the tyrosine kinase protein fragment of human RET [28]. Anti-phospho905 is a phospho-specific polyclonal antibody recognizing RET proteins phosphorylated at Y905 and anti-phospho1062 is a phospho-specific polyclonal antibody recognizing RET proteins phosphorylated at Y1062 [13]. Secondary antibodies coupled to horseradish peroxidase were from Santa Cruz Biotechnology.

### Growth Curves

RAT1, RAT RET/C634R and RAT RET/M918T cells (10,000/dish), Nthy-ory-3-1 (10,000/dish), TPC1 (10,000/dish), and MZ-CRC-1 (100,000/dish) and TT (200,000/dish) were seeded in 60-mm dishes. Fibroblasts were kept in medium supplemented with 5% fetal calf serum. Human cells were kept in 2% (TPC1), 5% (Nthy-ori-3-1), 20% (TT) or 10% (MZ-CRC-1) fetal calf serum. The day after plating, different concentrations of ALW-II-41-27, XMD15-44 and HG-6-63-01 or vehicle were added to the medium and refreshed every 2–3 days. Cells were counted every 1–2 (fibroblasts) or 2–3 (human cell lines) days. Ba/F3 cells were kept in 10% fetal calf serum in 6-well dishes (200,000/well) and counted daily for 4 consecutive days, changing media every 2 days.

### Statistical analysis

To compare cell growth, we performed unpaired Student’s *t* test using the Instat software program (Graphpad Software Inc). *P* values were two-sided, and differences were considered statistically significant at *P* <.02. IC_50_ doses were calculated through a curve fitting analysis from last day values using the PRISM software program (Graphpad Software Inc).

## Results

### Identification of three novel RET protein kinase inhibitors

**Fig 1:**
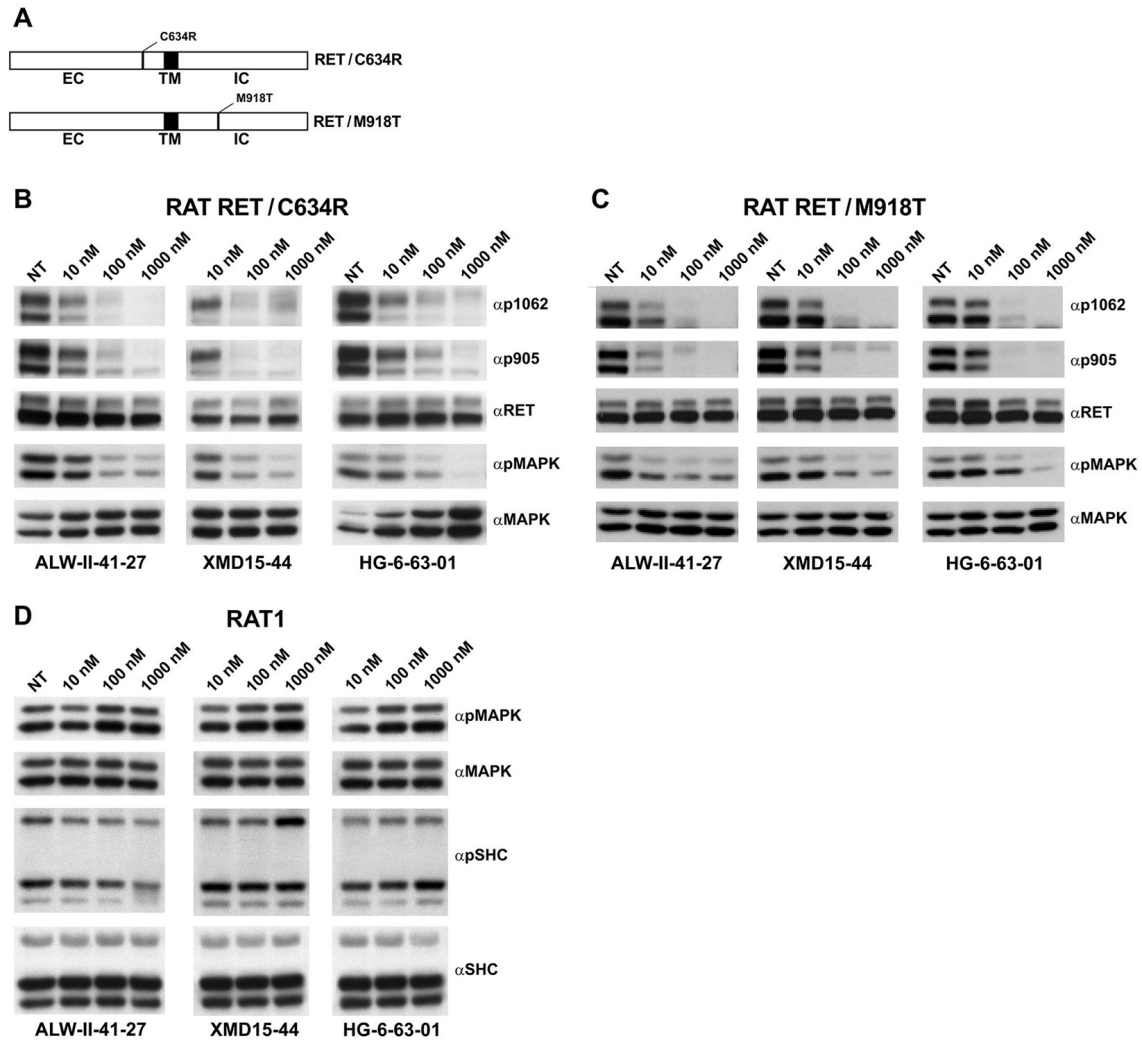
Inhibition of RET/C634R and RET/M918T phosphorylation and downstream signaling by ALW-II-41-27, XMD15-44 and HG-6-63-01. A) Schematic representation of RET/C634R and RET/M918T proteins. EC: extracellular domain; IC: intracellular domain; TM: transmembrane domain. B-D) Serum-starved RAT1 RET/C634R and RAT1 RET/M918T or parental RAT1 cells were treated for 2 hr with the indicated concentrations of ALW-II-41-27, XMD15-44 and HG-6-63-01. 50 μg of total cell lysates were subjected to immunoblotting with phospho-Y1062 (αp1062), phospho-Y905 (αp905) RET antibodies, phospho-MAPK (αpMAPK) or phospho-SHC (αpSHC) antibodies. The blots were normalized using anti-RET (αRET), anti-MAPK (αMAPK) and anti-SHC (αSHC) antibodies.

In order to identify new RET kinase inhibitors, we mined our in-house kinome wide kinase selectivity profiling database [17, 21, 23, 24]. We picked a small collection of 22 structurally diverse kinase inhibitors that possessed RET binding capacity and tested their effects in NIH3T3 fibroblasts stably expressing oncogenic RET/C634Y and RET/M918T proteins. Upon 2 hr drug treatment at 10, 100 and 1,000 nM, RET autophosphorylation and intracellular signaling to MAPK (p42/44 ERK) was examined by Western blotting with phospho-specific antibodies. This resulted in the identification of three compounds, ALW-II-41-27, XMD15-44 and HG-6-63-01, displaying strong (> 30%) inhibition at 10 nM of both RET/C634Y and RET/M918T proteins (data not shown). RET inhibition was confirmed in RAT1 fibroblasts stably expressing RET/C634R and RET/M918T (Fig 1A–1C). Of note, ALW-II-41-27, XMD15-44 and HG-6-63-01 did not affect SHC and MAPK phosphorylation in parental RAT1 cells (Fig 1D).

**Fig 2:**
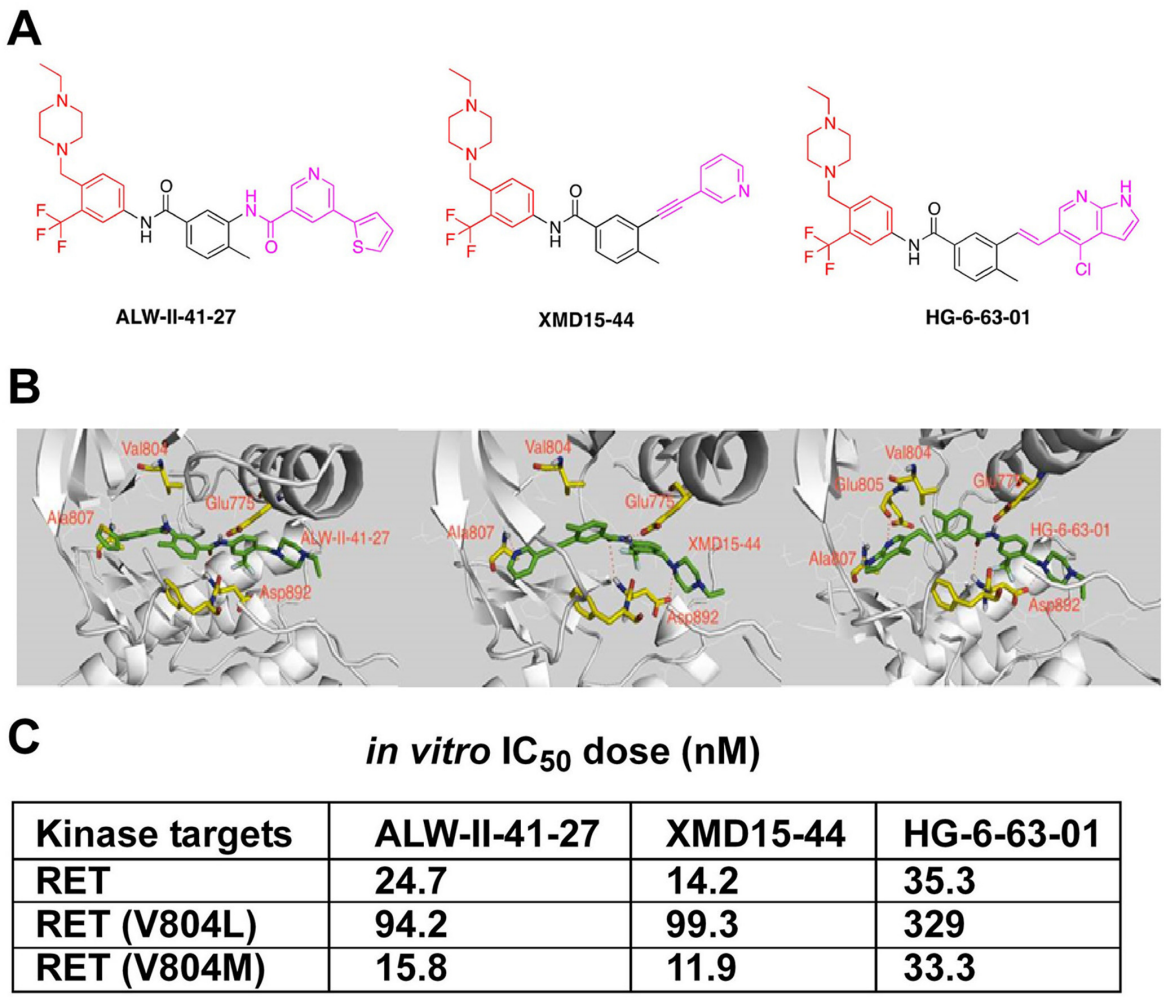
ALW-II-41-27, HG-6-63-01 and XMD15-44 chemical structure. A) Chemical structure of the studied compounds: red colour indicates the “tail”, black colour indicates “linker”, and pink colour indicates the “head” part in the binding. B) Modelling of ALW-II-41-27 (left), XMD15-44 (middle) and HG-6-63-01(right) binding to the DFG-out conformation of RET. C) IC_50_ dose (nM) of ALW-II-41-27, XMD15-44 and HG-6-63-01 for wt RET, RET/V804L and RET/V804M *in vitro* kinase activity measured by Invitrogen SelectScreen assay.

Structurally, ALW-II-41-27, XMD15-44 and HG-6-63-01 were all typical type II inhibitors [21–24]. Noteworthy, these three compounds possessed the same “linkers” (para-methyl benzylamide) and “tails” (3-trifluoromethyl-4-methylpiperazinephenyl) but they differ in the “head” portion that featured a nicotinamide (ALW-II-41-27), a pyrrolopyridine (HG-6-63-01) or a pyridine (XMD15-44) (Fig 2A). Molecular modelling suggested that they all shared the same binding mode to inactive conformation of RET (DFG-out) as expected for type II TKIs (Fig 2B) [17]. Indeed, the modelled conformation agrees well with their crystallographically determined structures in complex with ABL and EPH kinases [21, 24].

Though more rarely than C634 and M918 mutations, other mutations targeting RET residues in the tyrosine kinase domain can cause familial or sporadic MTC [1, 2]. We tested the activity of ALW-II-41-27, XMD15-44 and HG-6-63-01 towards various kinase domain-mutated RET oncoproteins. RET/L790F, RET/V804M and RET/S891A showed a sensitivity to ALW-II-41-27, XMD15-44 and HG-6-63-01 comparable to that of RET/C634R and RET/M918T proteins (S1 Fig). Instead, E768D, A883F and V804L RET mutants were less efficiently inhibited by the three compounds (S1 Fig). Of note, RET A883 residue is located in the VI Hanks domain adjacent to RET activation loop and it corresponds to G372 residue in ABL kinase whose mutation has been isolated in imatinib-resistant patients [33]. V804 corresponds to RET gatekeeper residue (T315 in ABL) in the ATP-binding pocket and was shown to mediate RET resistance to several kinase inhibitors when mutated to Methionine or Leucine [18, 20]. Characterization of the three compounds in biochemical kinase assays using the Invitrogen Selectscreen indicated that they are equally potent against both wild type RET and RET/V804M. However, RET/V804L mutants, likely because bulky nature of the leucine residue, featured 4–10 fold increased IC_50_ compared to wt kinase (Fig 2C).

ALW-II-41-27 has been previously reported as a potent EPH family kinase inhibitor [21, 22]; XMD15-44 has been characterized as a potent ABL inhibitor and HG-6-63-01 was developed as a general type II kinase inhibitor [23, 24]. Kinome wide selectivity profiling indicates that the three compounds have multiple targets in addition to RET (S2 Fig and S1 Table).

### Inhibition of RET point mutants-mediated cell growth by ALW-II-41-27, XMD15-44 and HG-6-63-01

**Fig 3:**
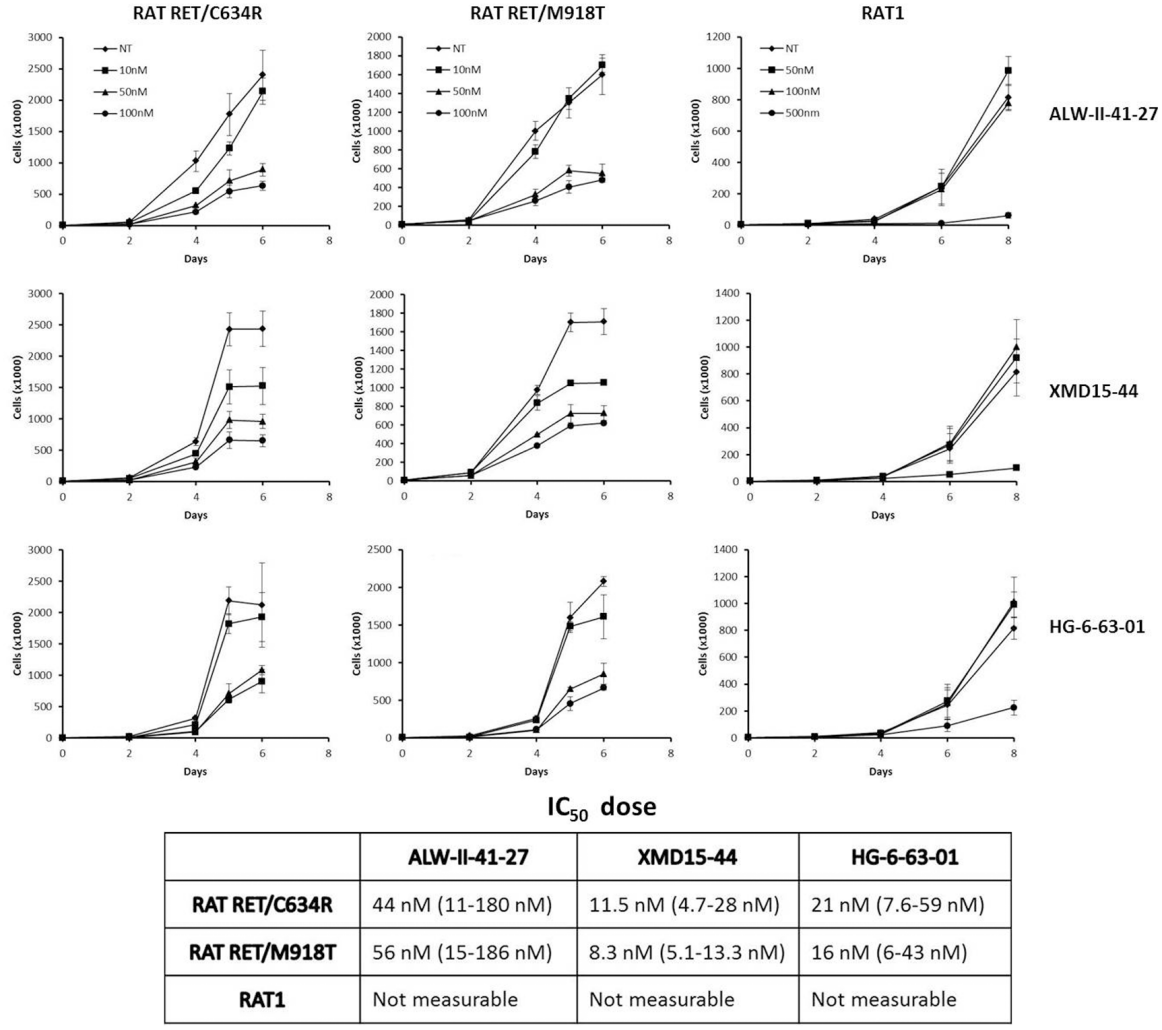
Growth inhibitory effects of ALW-II-41-27, XMD15-44 and HG-6-63-01 in RAT1 cells transformed by RET/C634R and RET/M918T. Top) Parental or RET/C634R and RET/M918T RAT1 cells were incubated in low serum with vehicle (NT: not treated) or the indicated concentrations of ALW-II-41-27, XMD15-44 and HG-6-63-01 and counted at the indicated time points. Data are the mean ± SD of two experiments performed in triplicate. Bottom) Growth inhibition IC_50_ of ALW-II-41-27, XMD15-44 and HG-6-63-01 for the different cell lines; 95% confidence intervals (CI) are indicated in brackets.

Expression of RET point mutants RET/C634R and RET/M918T stimulates RAT1 cells proliferation in low serum [29]. ALW-II-41-27, XMD15-44 and HG-6-63-01 reduced proliferation of RAT1 expressing RET/C634R or RET/M918T with an IC_50_ ≤ 56 nM (Fig 3). Parental RAT1 cell growth was affected only at one log higher dose.

### Inhibition of oncogenic RET rearrangements by ALW-II-41-27, XMD15-44 and HG-6-63-01

We tested compounds activity against most common RET-derived fusion oncogenes, including RET/PTC1 (CCDC6-RET), RET/PTC3 (NCOA4-RET) and KIF5B-RET. HEK293 cells were transiently transfected with the various RET constructs, and, after 48 hours, treated with compounds for 2 hr and harvested. ALW-II-41-27, XMD15-44 and HG-6-63-01 efficiently inhibited phosphorylation of the tested RET-derived chimeric proteins (S3 Fig).

The murine pro–B cell line Ba/F3 requires IL-3 for proliferation and survival [34]. RET/PTC3 expression made Ba/F3 cells independent on IL-3 for proliferation and treatment with ALW-II-41-27 blunted RET/PTC3-driven, but not IL-3-driven, proliferation (S3 Fig).

### Inhibition of endogenous RET oncoproteins in thyroid cancer cell lines by ALW-II-41-27, XMD15-44 and HG-6-63-01

**Fig 4:**
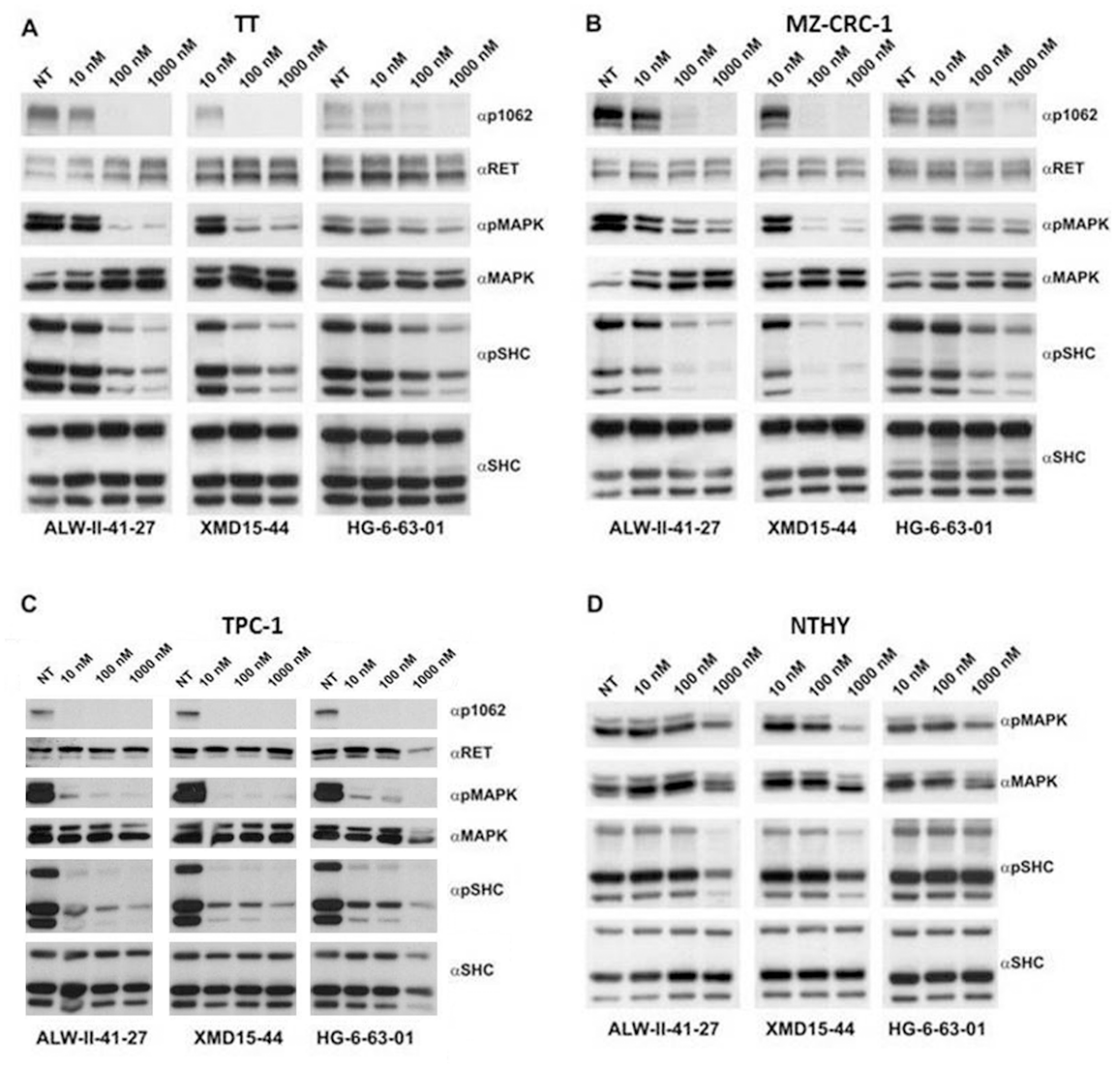
Inhibition of RET phosphorylation and signaling by ALW-II-41-27, XMD15-44 and HG-6-63-01 in RET mutant thyroid carcinoma cell lines. Serum-starved human TT (A) and MZ-CRC-1 (B) (MTC), TPC1 (PTC) (C), and non-transformed Nthy-ory-3-1 (NTHY) (D) cell lines were treated for 2 hr with indicated concentrations of ALW-II-41-27, HG-6-63-01 and XMD15-44. 50 μg of total cell lysates were subjected to immunoblotting with αp1062 RET antibodies, phospho-MAPK (αpMAPK) and phospho-SHC (αpSHC) antibodies. The blots were normalized using anti-RET (αRET), anti-MAPK (αMAPK) and anti-SHC (αSHC).

We selected TT and MZ-CRC-1 cells, that derive from human MTC harbouring the RET/C634W or RET/M918T mutation, respectively, and TPC1 cells, that derive from human PTC bearing the RET/PTC1 (CCDC6-RET) rearrangement [30–32]. Cells were treated with ALW-II-41-27, XMD15-44 and HG-6-63-01 at 10, 100 and 1,000 nM concentration and RET, SHC and MAPK phosphorylation was analysed. Concentrations as low at 10 nM of all three drugs inhibited RET autophosphorylation as well as SHC and MAPK activation by 20–40% of control (Fig 4A–4C). Importantly, at the used doses, ALW-II-41-27, XMD15-44 and HG-6-63-01 did not exert any detectable effects on SHC and MAPK phosphorylation in non-transformed thyroid follicular Nthy-ori-3-1 cells, used as control (Fig 4D).

**Fig 5:**
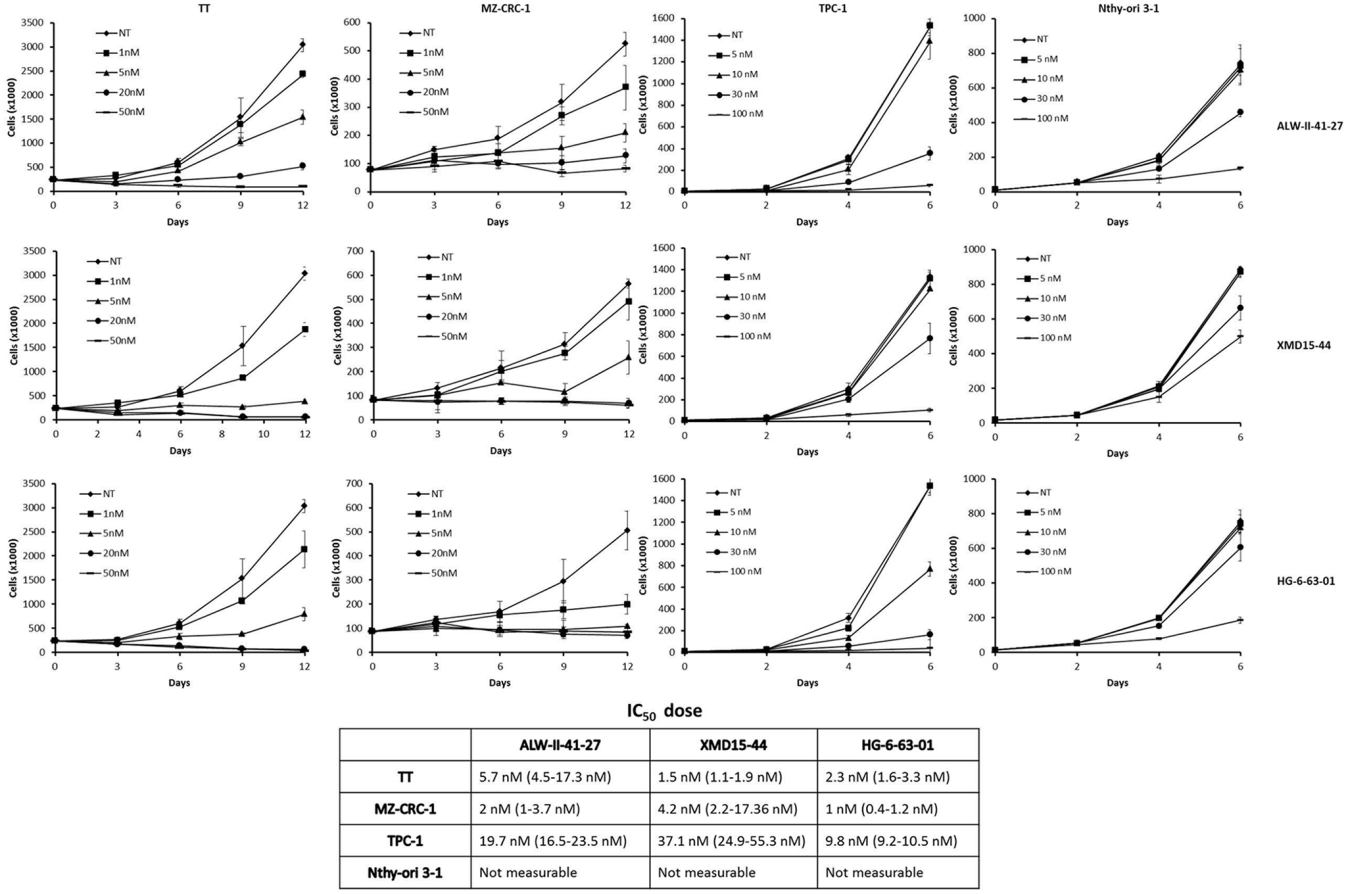
ALW-II-41-27, XMD15-44 and HG-6-63-01-mediated inhibition of proliferation of RET mutant thyroid carcinoma cell lines. Top) TT, MZ-CRC-1, TPC1 and Nthy-ori-3-1 cell lines were incubated with vehicle (NT: not treated) or the indicated concentrations of ALW-II-41-27, XMD15-44 and HG-6-63-01 and counted at the indicated time points. Data are the mean ± SD of two experiments performed in triplicate. Bottom) Growth inhibition IC_50_ of ALW-II-41-27, XMD15-44 and HG-6-63-01 for the different cell lines: 95% CI are indicated in brackets.

We analysed proliferation rate of TT, MZ-CRC-1 and TPC1 cells treated with the three compounds compared to control Nthy-ori-3-1 cells. Serum concentration was kept at 2% for TPC1, 20% for TT and 10% MZ-CRC-1 cells. ALW-II-41-27, XMD15-44 and HG-6-63-01 inhibited proliferation of RET mutant medullary thyroid carcinoma cells with an IC_50_ of 1.0–5.7 nM. Proliferation of papillary thyroid carcinoma (TPC1) cells was less strongly inhibited (IC_50_ of 9.8–37.1 nM). Proliferation of RET mutation negative control cells (Nthy-ori-3-1) started being affected at 30 nM dose and was clearly inhibited at 100 nM of the three compounds (Fig 5).

VEGFRII inhibition is believed to contribute, based on its anti-angiogenic effect, to clinical activity of approved RET inhibitors such as vandetanib and cabozantinib [14, 15]. According to kinome scanning (S1 Table), we could show, by Western blotting analysis, that ALW-II-41-27, XMD15-44 and HG-6-63-01 were able to inhibit VEGFRII phosphorylation in living TT cells (S4 Fig).

## Discussion

*RET* oncogenic conversion is a hallmark of several human cancers, including papillary and medullary thyroid carcinoma, lung adenocarcinoma and chronic myelomonocytic leukemia. In this light, RET kinase appears an attractive molecular target for anti-cancer therapy. Several anti-RET TKIs have been identified and vandetanib and cabozantinib have been recently approved for locally advanced or metastatic medullary thyroid carcinoma treatment [14, 15]. Both compounds are multitarget kinase inhibitors able to inhibit kinases other than RET, including VEGFRII (vandetanib and cabozantinib), EGFR (vandetanib) and MET (cabozantinib) [11, 12, 14, 15].

Here, we applied a structure-guided screen in order to identify novel RET TKIs. This screening resulted in the identification of ALW-II-41-27, XMD15-44, and HG-6-63-01 as novel and potent RET TKIs. Molecular modelling suggests that ALW-II-41-27, XMD15-44, and HG-6-63-01 recognize the ‘DFG-out’ conformation, consistent with being designed as type II kinase inhibitors. The three drugs impaired phosphorylation and signalling of various RET oncogenic mutants at nanomolar concentrations. They blunted proliferation of RET/C634R and RET/M918T-transformed fibroblasts and of RET mutant thyroid cancer cells. Of note, the three compounds bound to the RET kinase bearing V804M mutation that instead are refractory to vandetanib and cabozantinib [19, 20], whilst V804L mutation caused a 5–10 fold increase of the IC_50_ dose of the three drugs. This could be explained by the bulky nature of Leucine that may interfere with drug binding. On the other hand, differently from vandetanib, whose activity was not affected by mutations in RET kinase activation loop, RET inhibitory effect of ALW-II-41-27, XMD15-44, and HG-6-63-01 was impaired secondary to A883F mutation targeting the RET activation loop, which is consistent with their type II binding mode.

The newly identified RET TKIs shared a common structure with same “linkers” (para-methyl benzylamide) and “tails” (3-trifluoromethyl-4-methylpiperazinephenyl). We and others recently reported that ponatinib, another type II TKI, potently inhibits RET and its gate-keeper mutants [20, 35]. Ponatinib has the same linker and tail as ALW-II-41-27, HG-6-63-01 and XMD15-44 [36]. These findings suggest that this structure may efficiently bind to RET DFG-out conformation.

In conclusion, ALW-II-41-27, HG-6-63-01 and XMD15-44 represent novel lead compounds able to efficiently inhibit RET. However, given their broad kinase selectivity, it will be important their further optimization to develop clinically-relevant agents against RET.

## Supporting Information

S1 Fig Inhibition of phosphorylation of RET kinase domain mutants by ALW-II-41-27, XMD15-44 and HG-6-63-01. Top: Schematic representation of RET mutant proteins. EC: extracellular domain; IC: intracellular domain; TM: transmembrane domain. Serum-starved RAT1 cells expressing the indicated RET mutants were treated for 2 hr with indicated concentrations of ALW-II-41-27, XMD15-44 and HG-6-63-01. 50 μg of total cell lysates were subjected to immunoblotting with anti-phospho-Y1062 (αp1062) and-Y905 (αp905) RET antibodies. The blots were normalized using anti-RET.
(JPG)

S2 Fig Kinome wide selectivity profiling of RET inhibitors. Data were generated with DiscoveRx Treespot Version 4. Red dots indicate more than 99% of binding at 10 μM concentration of drug compared to DMSO control. S-score (1) indicated the selectivity when threshold was set at ≥99% inhibition. The size of the red circles is proportional to the strength of the binding, e.g. large circles imply high affinity.
(JPG)

S3 Fig Inhibition of RET/PTC1 (CCDC6-RET), RET/PTC3 (NCOA4-RET) and KIF5B-RET phosphorylation by ALW-II-41-27, XMD15-44 and HG-6-63-01 in HEK293 cells. A) HEK293 cells were transiently transfected with RET/PTC1, RET/PTC3 and KIF5B-RET expressing vectors. After 36 hr from transfection, cells were serum-starved for 12 hr and then treated for 2 hr with the indicated concentrations of ALW-II-41-27, XMD15-44 and HG-6-63-01. 50 μg of total cell lysates were subjected to immunoblotting with phospho-Y1062 (αp1062) and phospho-Y905 (αp905) RET antibodies. The blots were normalized using anti-RET (αRET) antibody. B) Parental Ba/F3 and Ba/F3 NCOA4-RET cells were incubated with vehicle (NT: not treated) or the indicated concentrations of ALW-II-41-27 in complete medium and counted at different time points. Differently from Ba/F3 NCOA4-RET, parental Ba/F3 were grown in the presence of IL3. Data are the mean ± SD of two experiments performed in triplicate. Growth inhibition IC_50_ of ALW-II-41-27 for the different cell lines with 95% CI are indicated.
(JPG)

S4 Fig ALW-II-41-27, XMD15-44 and HG-6-63-01-mediated inhibition of VEGFRII in TT cells. Serum-starved TT cells were treated for 2 hr with indicated concentrations of ALW-II-41-27, XMD15-44 and HG-6-63-01. 50 μg of total cell lysates were subjected to immunoblotting with anti- phospho-VEGFRII (αpVEGFRII) antibody. The blots were normalized using anti-VEGFRII (αVEGFRII) antibody.
(JPG)

S1 Methods Synthetic procedure and characterization of HG-6-63-01. (DOC)

S1 Table XMD15-44, ALW-II-41-27 and HG-6-63-01 in KinomeScan kinase panel. (DOC)
