# Supplementary figures and images for: Identification of Novel Small Molecule Inhibitors of Oncogenic RET Kinase
Source: PLoS One. 2015 Jun 5;10(6):e0128364. doi: 10.1371/journal.pone.0128364 (PMC4457528; doi:10.1371/journal.pone.0128364)

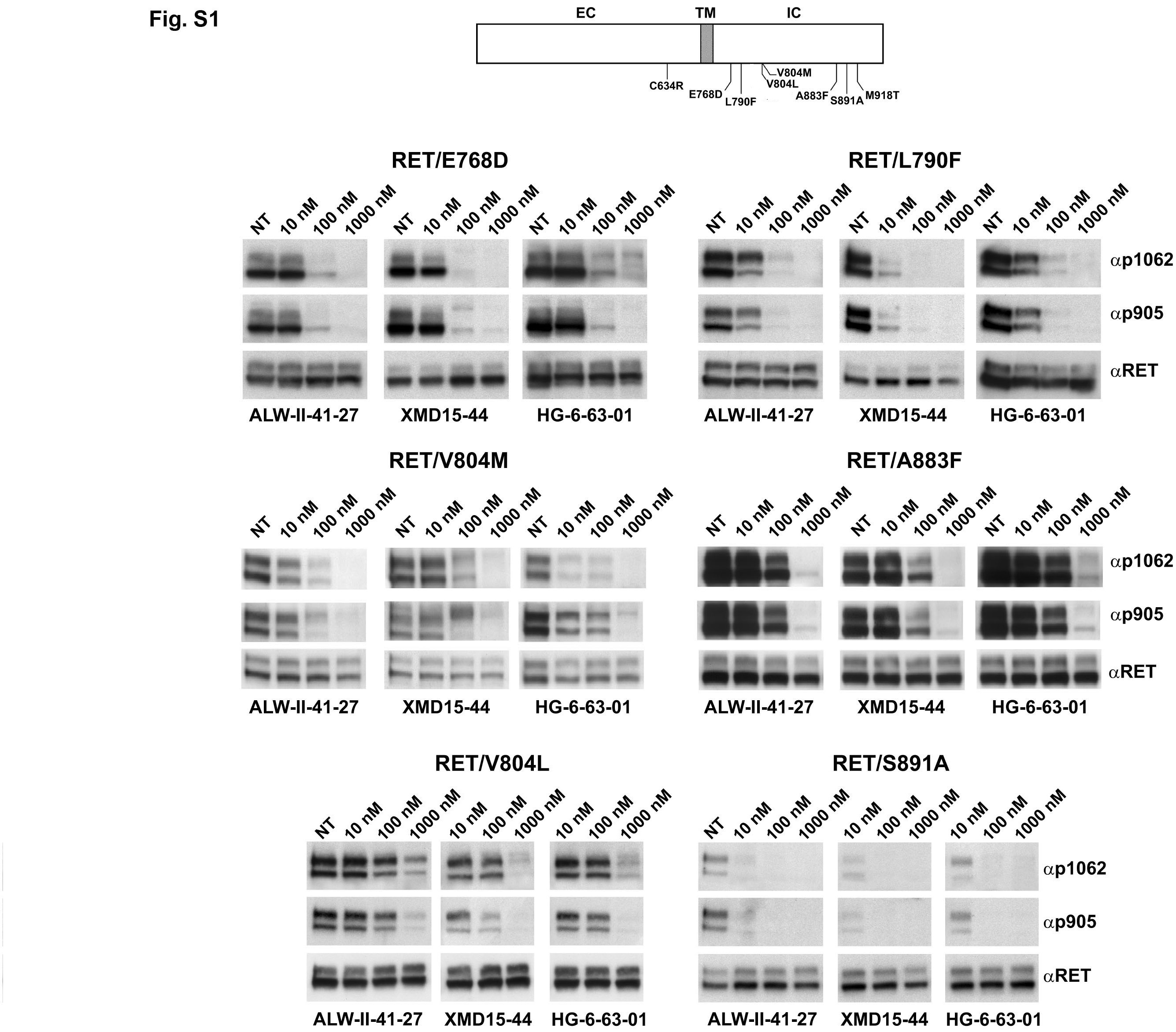

Supplement: S1 Fig — Top: Schematic representation of RET mutant proteins. EC: extracellular domain; IC: intracellular domain; TM: transmembrane domain. Serum-starved RAT1 cells expressing the indicated RET mutants were treated for 2 hr with indicated concentrations of ALW-II-41-27, XMD15-44 and HG-6-63-01. 50 μg of total cell lysates were subjected to immunoblotting with anti-phospho-Y1062 (αp1062) and-Y905 (αp905) RET antibodies. The blots were normalized using anti-RET. (JPG) [file pone.0128364.s001.jpg]

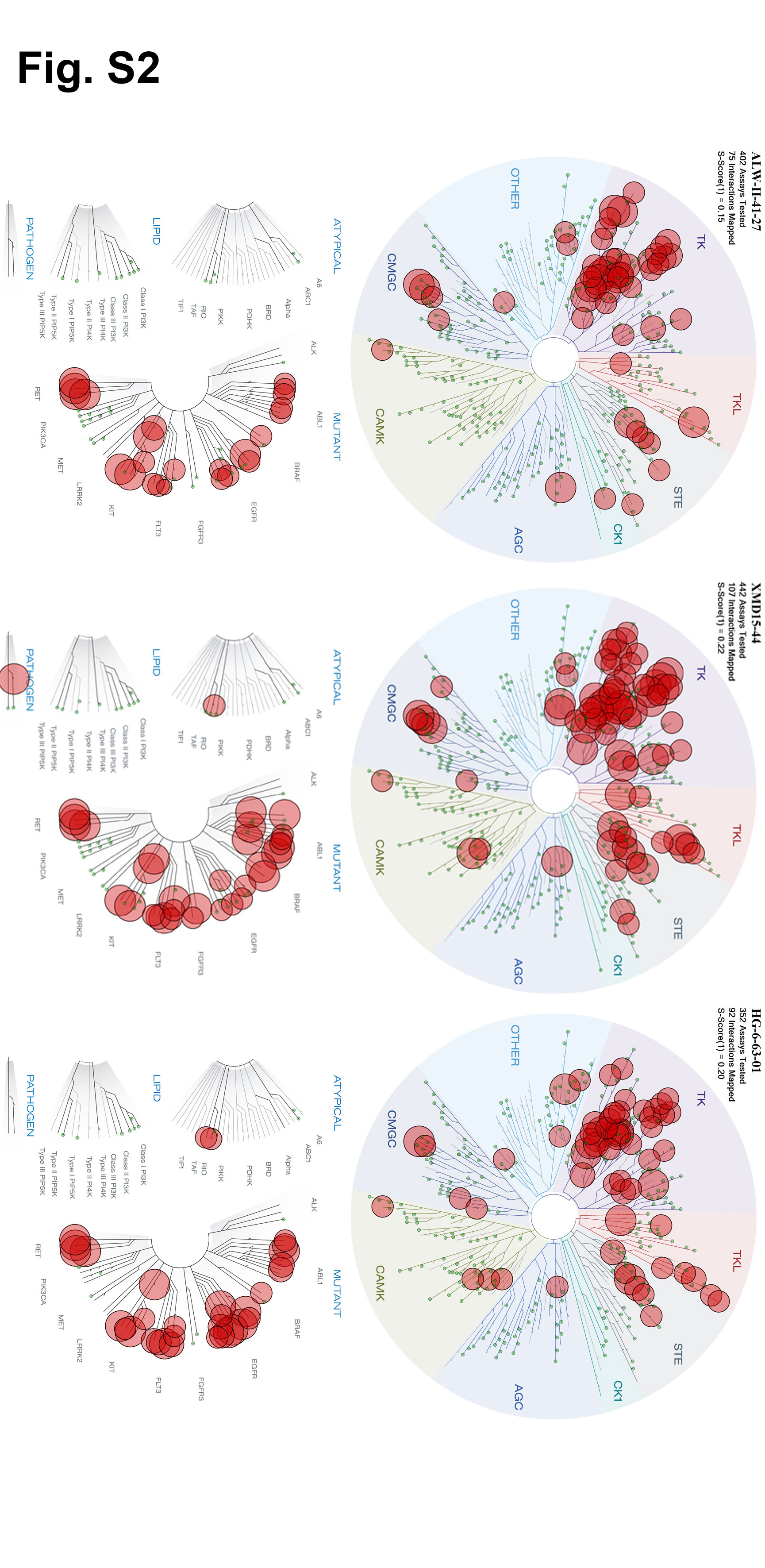

Supplement: S2 Fig — Data were generated with DiscoveRx Treespot Version 4. Red dots indicate more than 99% of binding at 10 μM concentration of drug compared to DMSO control. S-score (1) indicated the selectivity when threshold was set at ≥99% inhibition. The size of the red circles is proportional to the strength of the binding, e.g. large circles imply high affinity. (JPG) [file pone.0128364.s002.jpg]

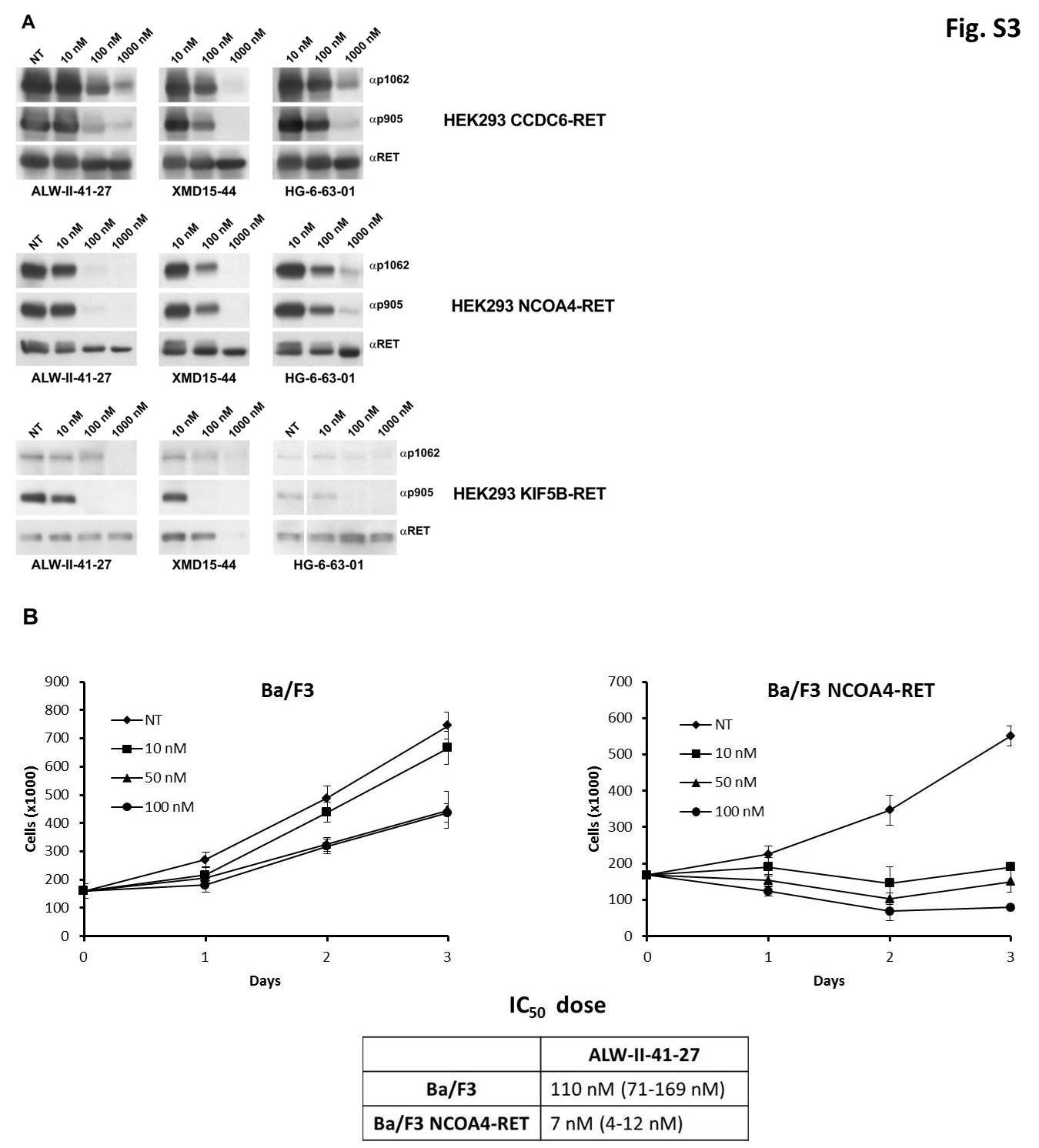

Supplement: S3 Fig — A) HEK293 cells were transiently transfected with RET/PTC1, RET/PTC3 and KIF5B-RET expressing vectors. After 36 hr from transfection, cells were serum-starved for 12 hr and then treated for 2 hr with the indicated concentrations of ALW-II-41-27, XMD15-44 and HG-6-63-01. 50 μg of total cell lysates were subjected to immunoblotting with phospho-Y1062 (αp1062) and phospho-Y905 (αp905) RET antibodies. The blots were normalized using anti-RET (αRET) antibody. B) Parental Ba/F3 and Ba/F3 NCOA4-RET cells were incubated with vehicle (NT: not treated) or the indicated concentrations of ALW-II-41-27 in complete medium and counted at different time points. Differently from Ba/F3 NCOA4-RET, parental Ba/F3 were grown in the presence of IL3. Data are the mean ± SD of two experiments performed in triplicate. Growth inhibition IC50 of ALW-II-41-27 for the different cell lines with 95% CI are indicated. (JPG) [file pone.0128364.s003.jpg]

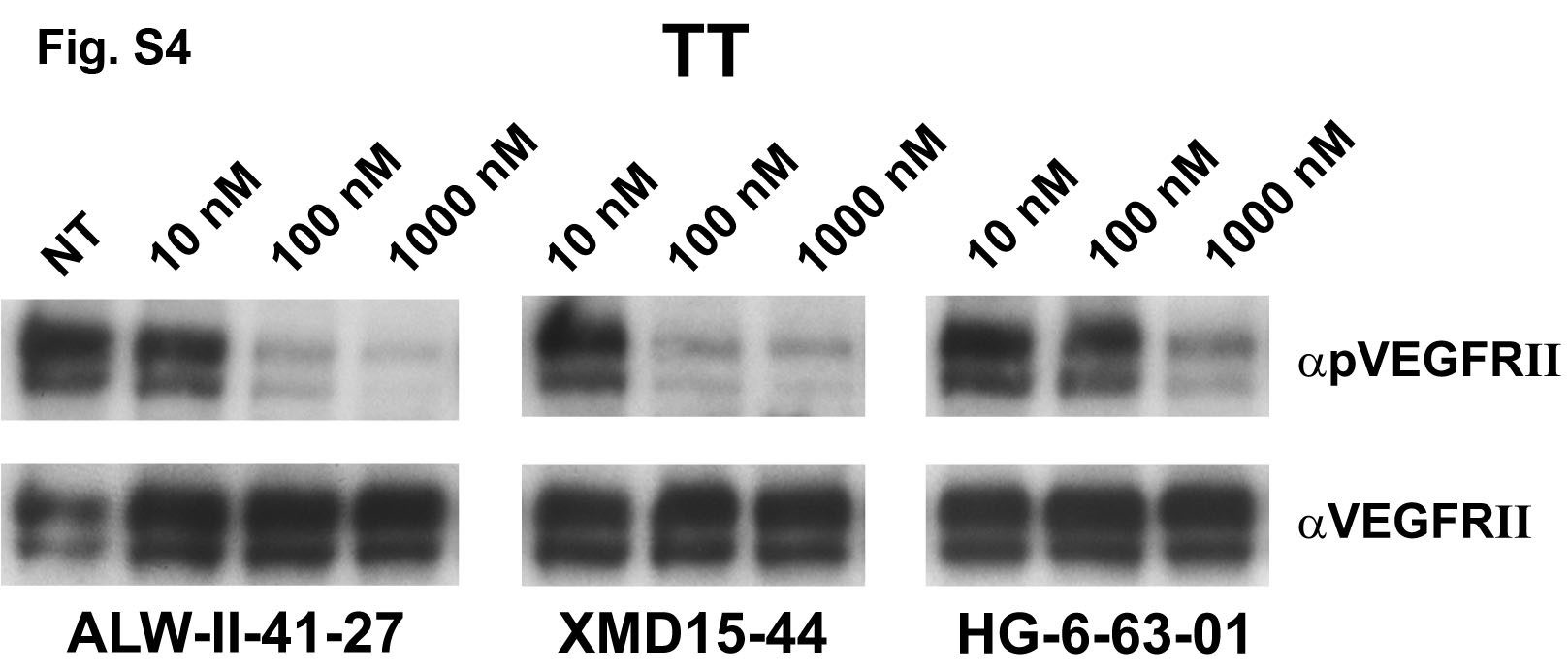

Supplement: S4 Fig — Serum-starved TT cells were treated for 2 hr with indicated concentrations of ALW-II-41-27, XMD15-44 and HG-6-63-01. 50 μg of total cell lysates were subjected to immunoblotting with anti- phospho-VEGFRII (αpVEGFRII) antibody. The blots were normalized using anti-VEGFRII (αVEGFRII) antibody. (JPG) [file pone.0128364.s004.jpg]
