# Supplementary material for: Identification of Novel Small Molecule Inhibitors of Oncogenic RET Kinase
Source: PLoS One. 2015 Jun 5;10(6):e0128364. doi: 10.1371/journal.pone.0128364 (PMC4457528; doi:10.1371/journal.pone.0128364)
Supplement: S1 Methods — (DOC) [file pone.0128364.s005.doc]

**Title: Identification of novel small molecule inhibitors of oncogenic RET kinase.**

**Supplemental Informations**

**Supplemental Methods**

**Experimental procedures for HG-6-63-01 synthesis and compound characterization**

**General ** All reactions were monitored by thin layer chromatography (TLC) with 0.25 mm E. Merck pre-coated silica gel plates (60 F_254_) and Waters LCMS system (Waters 2489 UV/Visible Detector, Waters 3100 Mass, Waters 515 HPLC pump, Waters 2545 Binary Gradient Module, Waters Reagent Manager, Waters 2767 Sample Manager) using SunFire^TM^ C18 column (4.6 x 50 mm, 5 mm particle size): solvent gradient = 97% A at 0 min, 0% A at 5 min; solvent A = 0.035% TFA in Water; solvent B = 0.035% TFA in Acetonitrile; flow rate : 3.0 mL/min. Purification of reaction products was carried out by flash chromatography using CombiFlash®Rf with Teledyne Isco Redi*Sep®*Rf High Performance Gold or Silicycle Silia*Sep*^TM^ High Performance columns (4 g, 12 g, 24 g, 40 g or 80 g). The purity of all compounds was over 95% and was analyzed with Waters LCMS system. ^1^H NMR and ^13^C NMR spectra were obtained using a Varian Inova-600 (600 MHz for ^1^H, and 125 MHz for ^13^C) spectrometer. Chemical shifts are reported relative to chloroform (*δ* = 7.24) for ^1^H NMR or dimethyl sulfoxide (*δ* = 2.50) for ^1^H NMR and dimethyl sulfoxide (*δ* = 39.51) for ^13^C NMR. Data are reported as (*br* = broad, *s* = singlet, *d* = doublet, *t* = triplet, *q* = quartet, *m* = multiplet).

**4-chloro-1-((2-(trimethylsilyl)ethoxy)methyl)-1H-pyrrolo[2,3-b]pyridine-5-carbaldehyde**

To a solution of 4-chloro-1H-pyrrolo[2,3-b]pyridine-5-carbaldehyde (500 mg, 2.78 mmol) in THF (9 mL) was added NaH (136 mg, 3.42 mmol) at 0 ^o^C. After 10 minutes, SEMCl (0.59 mL. 3.33 mmol) was slowly added to the reaction mixture at 0^o^C. The reaction mixture was warmed to room temperature and stirred for 2 hours after which, it was partitioned between ethyl acetate and water. The organic layer was separated and the aqueous layer was extracted with ethyl acetate. The combined organic extracts were washed with brine, dried over MgSO_4_, filtered through a pad of celite and concentrated under reduced pressure. The crude product was purified by flash chromatography using (5% to 20% Ethyl acetate/Hexane) as an eluent to afford title compound (810 mg, 94% yield). Rt = 4.28, ^1^H NMR (600 MHz, CDCl_3_) *δ* 10.62 (*s*, 1H), 8.90 (*s*, 1H), 7.52 (*d*, *J* = 3.6 Hz, 1H), 6.84 (*d*, *J* = 4.2 Hz, 1H), 5.76 (*s*, 2H), 3.60 (*t*, *J* = 8.4 Hz, 2H), 0.97 (*t*, *J* = 8.4 Hz, 2H), 0 (*s*, 9H), ^1^H NMR 600 MHz (DMSO-*d_6_*) *δ* 10.40 (*s*, 1H), 8.73 (*s*, 1H), 7.92 (*d*, *J* = 4.2 Hz, 1H), 6.82 (*d*, *J* = 4.2 Hz, 1H), 5.68 (*s*, 2H), 3.52 (*t*, *J* = 8.4 Hz, 2H), 0.82 (*t*, *J* = 8.4 Hz, 2H), -0.12 (*s*, 9H); ^13^C NMR 125 MHz (DMSO-*d_6_*) *δ* 188.80, 149.51, 145.10, 138.17, 132.21, 121.29, 119.23, 100.18, 72.89, 65.74, 17.06, -1.53; MS *m/z* : 311.31 [M+1].

**4-chloro-1-((2-(trimethylsilyl)ethoxy)methyl)-5-vinyl-1H-pyrrolo[2,3-b]pyridine**

To a solution methyltriphenylphosphonium iodide (782 mg, 1.93 mmol) in dried THF (5 mL) was added 2.5 M *n*-BuLi in Hexane (0.74 mL, 1.85 mmol) at –78 ^o^C. After 10 minutes, 4-chloro-1-((2-(trimethylsilyl)ethoxy)methyl)-1H-pyrrolo[2,3-b]pyridine-5-carbaldehyde (500 mg, 1.61 mmol) was added to the reaction mixture. The reaction mixture was warmed to room temperature spontaneously. After 2 hours, the reaction mixture was quenched with water and was partitioned between ethyl acetate and water. The organic layer was separated and the aqueous layer was extracted with ethyl acetate. The combined organic extracts were washed with brine, dried over MgSO_4_, celite filtered and concentrated under reduced pressure. The crude product was purified by flash chromatography using (5% to 15% Ethyl acetate/Hexane) as a solvent to afford title compound (380 mg, 76% yield). Rt = 4.88, ^1^H NMR (600 MHz, DMSO-*d_6_*) *δ* 8.59 (*s*, 1H), 7.74 (*d*, *J* = 3.6 Hz, 1H), 7.05 (*dd*, *J* = 18.0 Hz, *J* = 11.4 Hz, 1H), 6.59 (*d*, *J* = 3.6 Hz, 1H), 5.97 (*d*, *J* = 18.0 Hz, 1H), 5.62 (*s*, 2H), 5.45 (*d*, *J* = 11.4 Hz, 1H), 3.52 (*m*, 2H), 0.81 (*m*, 2H), -0.11 (*s*, 9H); MS *m/z* : 309.04 [M+1].

**N-(4-((4-ethylpiperazin-1-yl)methyl)-3-(trifluoromethyl)phenyl)-3-iodo-4-methylbenzamide**

3-iodo-4-methylbenzoic acid (1.5 g, 5.73 mmol) in thionyl chloride (15 mL) was heated under reflux for 1 h. The reaction mixture was cooled and the volatiles were concentrated under reduced pressure. The residue was co-evaporated with anhydrous toluene and dried under vacuum to afford 3-iodo-4-methylbenzoyl chloride which was used in next step reaction without further purification. To a solution of 4-((4-ethylpiperazin-1-yl)methyl)-3-(trifluoromethyl)aniline (1.48 g, 5.15 mmol) in anhydrous THF (15 mL) were added DIEA (4.98 mL, 28.63 mmol) and 3-iodo-4-methylbenzoyl chloride in anhydrous THF (15 mL) at 0 °C. The mixture was allowed to stir at room temperature for 3 h. The reaction mixture was quenched with cold water (30 mL) and extracted with ethyl acetate (3x50 mL). The organic layer was washed with brine, dried over MgSO_4_, filtered through a pad of celite and concentrated under reduced pressure. The residue was purified by column chromatography on silica gel (1:99 to 10:90, methanol/dichloromethane) to afford N-(4-((4-ethylpiperazin-1-yl)methyl)-3-(trifluoromethyl)phenyl)-3-iodo-4-methylbenzamide (2.4 g, 87% yield). Rt= 2.68, ^1^H NMR (DMSO-*d_6_*) *δ* 10.45 (*s*, 1H), 8.40 (*d*, *J* = 1.8 Hz, 1H), 8.16 (*d*, *J* = 2.4 Hz, 1H), 8.02 (*dd*, *J* = 1.8 Hz, *J* = 7.8 Hz, 1H), 7.90 (*d*, *J* = 1.8 Hz, *J* = 7.8 Hz, 1H), 7.69 (*d*, *J* = 8.4 Hz, 1H), 7.48 (*d*, *J* = 7.8 Hz, 1H), 3.54 (*s*, 2H), 2.48 (*m*, 2H), 2.42 (*s*, 3H), 2.31-2.40 (*m*, 8H), 0.98 (*t*, *J* = 7.2 Hz, 3H); ^13^C NMR 125 MHz (DMSO-*d_6_*) *δ* 166.95, 148.06, 141.21, 140.59, 136.64, 135.11, 134.22, 132.85, 130.58, 130.38, 128.32, 126.60, 120.40, 104.17, 60.50, 55.61, 55.31, 54.59, 30.66, 14.81; MS *m/z* : 532.23 [M+1].

**(E)-3-(2-(4-chloro-1-((2-(trimethylsilyl)ethoxy)methyl)-1H-pyrrolo[2,3-b]pyridin-5-yl)vinyl)-N-(4-((4-ethylpiperazin-1-yl)methyl)-3-(trifluoromethyl)phenyl)-4-methylbenzamide**

A mixture of N-(4-((4-ethylpiperazin-1-yl)methyl)-3-(trifluoromethyl)phenyl)-3-iodo-4-methylbenzamide (300 mg, 0.56 mmol), 4-chloro-1-((2-(trimethylsilyl)ethoxy)methyl)-5-vinyl-1H-pyrrolo[2,3-b]pyridine (191 mg, 0.62 mmol), Pd(OAc)_2_ (13 mg, 0.06 mmol), tri-*p*-tolylphosphine (26 mg, 0.08 mmol), and DIEA (294 mL, 1.13 mmol) in DMF (3 ml) was heated at 120 ℃ for 18 h after which, it was filtered with a pad of celite and partitioned between ethyl acetate and water. The organic layer was separated and the aqueous layer was extracted with ethyl acetate. The combined organic extracts were washed with brine, dried over MgSO_4_, celite filtered and concentrated under reduced pressure. The crude product was purified by flash on silica gel (1:99 to 7:93, methanol/dichloromethane) to afford title compound (310 mg, 77% yield). Rt= 3.62, ^1^H NMR (600 MHz, DMSO-*d_6_*) *δ* 10.53 (*s*, 1H), 8.85 (*s*, 1H), 8.27 (*d*, *J* = 1.8 Hz, 1H), 8.23 (*d*, *J* = 1.8 Hz, 1H), 8.06 (*dd*, *J* = 9.0 Hz, *J* = 1.8 Hz, 1H), 7.84 (*dd*, *J* = 7.8 Hz, *J* = 1.8 Hz, 1H), 7.78 (*d*, *J* = 3.6 Hz, 1H), 7.72 (*d*, *J* = 9.0 Hz, 1H), 7.61 (*d*, *J* = 16.8 Hz, 1H), 7.51 (*d*, *J* = 16.8 Hz, 1H), 7.42 (*d*, *J* = 8.4 Hz, 1H), 6.64 (*d*, *J* = 4.2 Hz, 1H), 5.65 (*s*, 2H), 3.55 (*m*, 4H), 2.51 (*s*, 3H), 2.44-2.42 (*m*, 10H), 0.98 (*t*, *J* = 7.2 Hz, 3H), 0.83 (*t*, *J* = 7.8 Hz, 2H), -0.093 (*s*, 9H), MS *m/z* : 712.52 [M+1].

**(E)-3-(2-(4-chloro-1H-pyrrolo[2,3-b]pyridin-5-yl)vinyl)-N-(4-((4-ethylpiperazin-1-yl)methyl)-3-(trifluoromethyl)phenyl)-4-methylbenzamide**

To a solution of (E)-3-(2-(4-chloro-1-((2-(trimethylsilyl)ethoxy)methyl)-1H-pyrrolo[2,3-b]pyridin-5-yl)vinyl)-N-(4-((4-ethylpiperazin-1-yl)methyl)-3-(trifluoromethyl)phenyl)-4-methylbenzamide (200 mg, 0.28mmol) in CH_2_Cl_2_ (2 mL) was added TFA (1 mL). The reaction mixture was stirred for 4 hour and the volatiles were concentrated under reduced pressure. To a solution of the resulting mixture in THF (1 mL) and MeOH (1 mL) was added LiOH•H_2_O (117 mg, 2.81 mmol) in water (1 mL). The reaction mixture was stirred for 4 hours at room temperature. The organic solvent was removed under reduced pressure and the aqueous layer was extracted with ethyl acetate. The organic extracts were washed with brine, dried over MgSO_4_, celite filtered and concentrated under reduced pressure. The crude product was purified by flash on silica gel (1:99 to 10:90, methanol/dichloromethane) to afford title compound (110 mg, 67% yield). Rt = 2.77, ^1^H NMR (600 MHz, DMSO-*d_6_*) *δ* 12.10 (*s*, 1H), 10.53 (*s*, 1H), 8.77 (*s*, 1H), 8.25 (*s*, 1H), 8.21 (*s*, 1H), 8.04 (*d*, *J* = 7.8 Hz, 1H), 7.82 (*d*, *J* = 8.4 Hz, 1H), 7.70 (*d*, *J* = 8.4 Hz, 1H), 7.57 (*s*, 2H), 7.54 (*s*, 1H), 7.50 (*s*, 1H), 7.39 (*d*, *J* = 7.2 Hz, 1H), 3.55 (*m*, 2H), 2.48 (*s*, 3H), 2.39 (*m*, 8H), 2.86 (*m*, 2H), 0.96 (*t*, *J* = 7.2 Hz, 3H). MS *m/z* : 582.48 [M+1].
