# Supplementary material for: Identification of Novel Small Molecule Inhibitors of Oncogenic RET Kinase
Source: PLoS One. 2015 Jun 5;10(6):e0128364. doi: 10.1371/journal.pone.0128364 (PMC4457528; doi:10.1371/journal.pone.0128364)
Supplement: S1 Table — (DOC) [file pone.0128364.s006.doc]

**Title: Identification of novel small molecule inhibitors of oncogenic RET kinase.**

**Supplemental Informations**

**Supplemental Table**

**Table: XMD15-44, ALW-II-41-27 and HG-6-63-01 in KinomeScan kinase panel**

Inhibitors were screened at a single concentration of 10 μM. At this concentration, a score of less than 10% implies that the false positive probability is less than 20% and that the *K_d_* value is most likely less than 1 μM; a score between 1 and 10% implies that the false positive probability is less than 10% and a score of less than 1% implies that the false positive probability is less than 5% and that the *K_d_* value is most likely less than 1 μM. Hits values higher than 30% were considered negative (no binding: nb).

| **XMD15-44** | |  | **ALW-II-41-27** | |  | **HG-6-63-01** | |
| --- | --- | --- | --- | --- | --- | --- | --- |
| **Kinase** | **score (%)** |  | **Kinase** | **score (%)** |  | **Kinase** | **score (%)** |
| **AAK1** | **nb** |  | **AAK1** | **nb** |  | **AAK1** | **27** |
| **ABL1(E255K)-phosphorylated** | **0.05** |  | **ABL1** | **0.05** |  | **ABL1** | **0.3** |
| **ABL1(F317I)-nonphosphorylated** | **8.8** |  | **ABL1(E255K)** | **7.2** |  | **ABL1(E255K)** | **1.3** |
| **ABL1(F317I)-phosphorylated** | **4** |  | **ABL1(F317I)** | **0.2** |  | **ABL1(F317I)** | **0.05** |
| **ABL1(F317L)-nonphosphorylated** | **0** |  | **ABL1(F317L)** | **0.2** |  | **ABL1(F317L)** | **0.1** |
| **ABL1(F317L)-phosphorylated** | **0.85** |  | **ABL1(H396P)** | **0.5** |  | **ABL1(H396P)** | **0.2** |
| **ABL1(H396P)-nonphosphorylated** | **0** |  | **ABL1(M351T)** | **0.55** |  | **ABL1(M351T)** | **0.1** |
| **ABL1(H396P)-phosphorylated** | **0** |  | **ABL1(Q252H)** | **0.25** |  | **ABL1(Q252H)** | **0.1** |
| **ABL1(M351T)-phosphorylated** | **2.6** |  | **ABL1(T315I)** | **0.7** |  | **ABL1(T315I)** | **0** |
| **ABL1(Q252H)-nonphosphorylated** | **6.4** |  | **ABL1(Y253F)** | **0.2** |  | **ABL1(Y253F)** | **0.1** |
| **ABL1(Q252H)-phosphorylated** | **0.7** |  | **ABL2** | **0.1** |  | **ABL2** | **0.1** |
| **ABL1(T315I)-nonphosphorylated** | **2.8** |  | **ACVR1** | **nb** |  | **ACVR1** | **nb** |
| **ABL1(T315I)-phosphorylated** | **2.6** |  | **ACVR1B** | **nb** |  | **ACVR1B** | **nb** |
| **ABL1(Y253F)-phosphorylated** | **0.15** |  | **ACVR2A** | **nb** |  | **ACVR2A** | **nb** |
| **ABL1-nonphosphorylated** | **0** |  | **ACVR2B** | **nb** |  | **ACVR2B** | **nb** |
| **ABL1-phosphorylated** | **0** |  | **ACVRL1** | **nb** |  | **ACVRL1** | **nb** |
| **ABL2** | **0.05** |  | **ADCK3** | **nb** |  | **ADCK3** | **nb** |
| **ACVR1** | **nb** |  | **ADCK4** | **nb** |  | **ADCK4** | **nb** |
| **ACVR1B** | **nb** |  | **AKT1** | **4.4** |  | **AKT1** | **nb** |
| **ACVR2A** | **nb** |  | **AKT2** | **nb** |  | **AKT2** | **nb** |
| **ACVR2B** | **nb** |  | **AKT3** | **20** |  | **AKT3** | **nb** |
| **ACVRL1** | **nb** |  | **ALK** | **nb** |  | **ALK** | **nb** |
| **ADCK3** | **nb** |  | **AMPK-alpha1** | **nb** |  | **AMPK-alpha1** | **18** |
| **ADCK4** | **nb** |  | **AMPK-alpha2** | **nb** |  | **AMPK-alpha2** | **nb** |
| **AKT1** | **nb** |  | **ANKK1** | **1.4** |  | **ANKK1** | **0.4** |
| **AKT2** | **30** |  | **ARK5** | **nb** |  | **ARK5** | **nb** |
| **AKT3** | **nb** |  | **ASK1** | **nb** |  | **AURKA** | **24** |
| **ALK** | **23** |  | **ASK2** | **nb** |  | **AURKB** | **6.6** |
| **AMPK-alpha1** | **1.2** |  | **AURKA** | **nb** |  | **AURKC** | **0.65** |
| **AMPK-alpha2** | **18** |  | **AURKB** | **nb** |  | **AXL** | **17** |
| **ANKK1** | **8.2** |  | **AURKC** | **nb** |  | **BIKE** | **1.2** |
| **ARK5** | **nb** |  | **AXL** | **nb** |  | **BLK** | **0.2** |
| **ASK1** | **nb** |  | **BIKE** | **nb** |  | **BMPR1A** | **nb** |
| **ASK2** | **12** |  | **BLK** | **0.1** |  | **BMPR1B** | **nb** |
| **AURKA** | **nb** |  | **BMPR1A** | **nb** |  | **BMPR2** | **8.2** |
| **AURKB** | **24** |  | **BMPR1B** | **nb** |  | **BMX** | **1.6** |
| **AURKC** | **13** |  | **BMPR2** | **nb** |  | **BRAF** | **1.2** |
| **AXL** | **2.9** |  | **BMX** | **11** |  | **BRAF(V600E)** | **0.2** |
| **BIKE** | **nb** |  | **BRAF** | **1.8** |  | **BRSK1** | **nb** |
| **BLK** | **0** |  | **BRAF(V600E)** | **0.55** |  | **BRSK2** | **nb** |
| **BMPR1A** | **nb** |  | **BRK** | **8.2** |  | **BTK** | **3.8** |
| **BMPR1B** | **nb** |  | **BRSK1** | **nb** |  | **CAMK1** | **nb** |
| **BMPR2** | **nb** |  | **BRSK2** | **nb** |  | **CAMK1D** | **nb** |
| **BMX** | **2.7** |  | **BTK** | **0.1** |  | **CAMK1G** | **nb** |
| **BRAF** | **0.05** |  | **CAMK1** | **nb** |  | **CAMK2A** | **nb** |
| **BRAF(V600E)** | **0.05** |  | **CAMK1D** | **nb** |  | **CAMK2B** | **nb** |
| **BRK** | **2.8** |  | **CAMK1G** | **nb** |  | **CAMK2D** | **nb** |
| **BRSK1** | **nb** |  | **CAMK2A** | **nb** |  | **CAMK2G** | **nb** |
| **BRSK2** | **nb** |  | **CAMK2B** | **nb** |  | **CAMK4** | **nb** |
| **BTK** | **2** |  | **CAMK2D** | **nb** |  | **CAMKK1** | **nb** |
| **CAMK1** | **nb** |  | **CAMK2G** | **nb** |  | **CAMKK2** | **nb** |
| **CAMK1D** | **nb** |  | **CAMK4** | **nb** |  | **CDC2L1** | **13** |
| **CAMK1G** | **nb** |  | **CAMKK1** | **33** |  | **CDC2L2** | **17** |
| **CAMK2A** | **nb** |  | **CAMKK2** | **nb** |  | **CDK11** | **3** |
| **CAMK2B** | **nb** |  | **CDC2L1** | **1.5** |  | **CDK2** | **nb** |
| **CAMK2D** | **nb** |  | **CDC2L2** | **1.8** |  | **CDK3** | **nb** |
| **CAMK2G** | **nb** |  | **CDK11** | **3.8** |  | **CDK5** | **nb** |
| **CAMK4** | **nb** |  | **CDK2** | **nb** |  | **CDK7** | **nb** |
| **CAMKK1** | **nb** |  | **CDK3** | **nb** |  | **CDK8** | **9.6** |
| **CAMKK2** | **7.8** |  | **CDK5** | **nb** |  | **CDK9** | **nb** |
| **CASK** | **nb** |  | **CDK7** | **26** |  | **CDKL2** | **0.5** |
| **CDC2L1** | **2.5** |  | **CDK8** | **20** |  | **CHEK1** | **25** |
| **CDC2L2** | **1.6** |  | **CDK9** | **nb** |  | **CHEK2** | **nb** |
| **CDC2L5** | **6.5** |  | **CDKL2** | **9.1** |  | **CIT** | **0.2** |
| **CDK11** | **5** |  | **CDKL3** | **9.8** |  | **CLK1** | **27** |
| **CDK2** | **10** |  | **CDKL5** | **nb** |  | **CLK2** | **nb** |
| **CDK3** | **24** |  | **CHEK1** | **nb** |  | **CLK3** | **nb** |
| **CDK4-cyclinD1** | **21** |  | **CHEK2** | **nb** |  | **CLK4** | **nb** |
| **CDK4-cyclinD3** | **nb** |  | **CIT** | **14** |  | **CSF1R** | **0** |
| **CDK5** | **10** |  | **CLK1** | **1.1** |  | **CSK** | **2.1** |
| **CDK7** | **1.6** |  | **CLK2** | **2.4** |  | **CSNK1A1L** | **nb** |
| **CDK8** | **8.2** |  | **CLK3** | **nb** |  | **CSNK1D** | **nb** |
| **CDK9** | **nb** |  | **CLK4** | **5.3** |  | **CSNK1E** | **15** |
| **CDKL1** | **nb** |  | **CSF1R** | **0** |  | **CSNK1G1** | **nb** |
| **CDKL2** | **1.4** |  | **CSK** | **0.65** |  | **CSNK1G2** | **nb** |
| **CDKL3** | **0.65** |  | **CSNK1A1L** | **nb** |  | **CSNK1G3** | **nb** |
| **CDKL5** | **12** |  | **CSNK1D** | **5** |  | **CSNK2A1** | **15** |
| **CHEK1** | **nb** |  | **CSNK1E** | **0.8** |  | **CSNK2A2** | **nb** |
| **CHEK2** | **nb** |  | **CSNK1G1** | **nb** |  | **DAPK1** | **nb** |
| **CIT** | **0** |  | **CSNK1G2** | **20** |  | **DAPK2** | **nb** |
| **CLK1** | **3.9** |  | **CSNK1G3** | **27** |  | **DAPK3** | **nb** |
| **CLK2** | **nb** |  | **CSNK2A1** | **nb** |  | **DCAMKL1** | **nb** |
| **CLK3** | **nb** |  | **CSNK2A2** | **nb** |  | **DCAMKL2** | **nb** |
| **CLK4** | **15** |  | **CTK** | **nb** |  | **DCAMKL3** | **2.5** |
| **CSF1R** | **0** |  | **DAPK1** | **nb** |  | **DDR1** | **0.1** |
| **CSK** | **0.15** |  | **DAPK2** | **nb** |  | **DDR2** | **0.8** |
| **CSNK1A1** | **nb** |  | **DAPK3** | **nb** |  | **DLK** | **5.5** |
| **CSNK1A1L** | **nb** |  | **DCAMKL1** | **nb** |  | **DMPK** | **nb** |
| **CSNK1D** | **29** |  | **DCAMKL2** | **nb** |  | **DMPK2** | **14** |
| **CSNK1E** | **nb** |  | **DCAMKL3** | **nb** |  | **DRAK1** | **0.35** |
| **CSNK1G1** | **nb** |  | **DDR1** | **0.15** |  | **DRAK2** | **1.6** |
| **CSNK1G2** | **nb** |  | **DDR2** | **12** |  | **DYRK1B** | **22** |
| **CSNK1G3** | **nb** |  | **DLK** | **28** |  | **EGFR** | **0.3** |
| **CSNK2A1** | **nb** |  | **DMPK** | **nb** |  | **EGFR(E746-A750del)** | **0.9** |
| **CSNK2A2** | **nb** |  | **DMPK2** | **nb** |  | **EGFR(G719C)** | **0.1** |
| **CTK** | **nb** |  | **DRAK1** | **nb** |  | **EGFR(G719S)** | **0.1** |
| **DAPK1** | **nb** |  | **DRAK2** | **31** |  | **EGFR(L747-E749del. A750P)** | **0** |
| **DAPK2** | **nb** |  | **DYRK1A** | **0.15** |  | **EGFR(L747-S752del. P753S)** | **0** |
| **DAPK3** | **nb** |  | **DYRK1B** | **12** |  | **EGFR(L747-T751del.Sins)** | **0** |
| **DCAMKL1** | **nb** |  | **DYRK2** | **18** |  | **EGFR(L858R)** | **0.4** |
| **DCAMKL2** | **nb** |  | **EGFR** | **1.3** |  | **EGFR(L861Q)** | **0** |
| **DCAMKL3** | **nb** |  | **EGFR(E746-A750del)** | **1.2** |  | **EGFR(S752-I759del)** | **0** |
| **DDR1** | **0.05** |  | **EGFR(G719C)** | **0.15** |  | **EPHA1** | **5.5** |
| **DDR2** | **1** |  | **EGFR(G719S)** | **0.5** |  | **EPHA2** | **0.25** |
| **DLK** | **9.8** |  | **EGFR(L747-E749del. A750P)** | **0.55** |  | **EPHA3** | **11** |
| **DMPK** | **nb** |  | **EGFR(L747-S752del. P753S)** | **3** |  | **EPHA4** | **0.55** |
| **DMPK2** | **nb** |  | **EGFR(L747-T751del.Sins)** | **1.8** |  | **EPHA5** | **3.3** |
| **DRAK1** | **nb** |  | **EGFR(L858R)** | **0.25** |  | **EPHA6** | **6.7** |
| **DRAK2** | **nb** |  | **EGFR(L858R.T790M)** | **nb** |  | **EPHA7** | **nb** |
| **DYRK1A** | **nb** |  | **EGFR(L861Q)** | **0** |  | **EPHA8** | **0.1** |
| **DYRK1B** | **nb** |  | **EGFR(S752-I759del)** | **3** |  | **EPHB1** | **0.7** |
| **DYRK2** | **nb** |  | **EPHA1** | **3.8** |  | **EPHB2** | **1.1** |
| **EGFR** | **3** |  | **EPHA2** | **0.15** |  | **EPHB3** | **11** |
| **EGFR(E746-A750del)** | **1.8** |  | **EPHA3** | **7.6** |  | **EPHB4** | **5.7** |
| **EGFR(G719C)** | **0.75** |  | **EPHA4** | **0** |  | **ERBB2** | **21** |
| **EGFR(G719S)** | **2.4** |  | **EPHA5** | **0.25** |  | **ERBB4** | **0.7** |
| **EGFR(L747-E749del. A750P)** | **1.2** |  | **EPHA6** | **28** |  | **ERK1** | **nb** |
| **EGFR(L747-S752del. P753S)** | **0.9** |  | **EPHA7** | **nb** |  | **ERK2** | **nb** |
| **EGFR(L747-T751del.Sins)** | **0.45** |  | **EPHA8** | **0.1** |  | **ERK3** | **nb** |
| **EGFR(L858R)** | **4** |  | **EPHB1** | **0.15** |  | **ERK4** | **nb** |
| **EGFR(L858R.T790M)** | **nb** |  | **EPHB2** | **0** |  | **ERK5** | **nb** |
| **EGFR(L861Q)** | **0.2** |  | **EPHB3** | **0.3** |  | **ERK8** | **nb** |
| **EGFR(S752-I759del)** | **0.9** |  | **EPHB4** | **0.55** |  | **FER** | **14** |
| **EGFR(T790M)** | **nb** |  | **EPHB6** | **1.2** |  | **FES** | **0.55** |
| **EIF2AK1** | **nb** |  | **ERBB2** | **11** |  | **FGFR1** | **0.7** |
| **EPHA1** | **1.2** |  | **ERBB3** | **nb** |  | **FGFR2** | **1.6** |
| **EPHA2** | **0.4** |  | **ERBB4** | **1.3** |  | **FGFR3** | **2.4** |
| **EPHA3** | **0.85** |  | **ERK1** | **nb** |  | **FGFR3(G697C)** | **2.4** |
| **EPHA4** | **0** |  | **ERK2** | **nb** |  | **FGFR4** | **0.2** |
| **EPHA5** | **1.2** |  | **ERK3** | **nb** |  | **FGR** | **0.35** |
| **EPHA6** | **0** |  | **ERK4** | **nb** |  | **FLT1** | **0.2** |
| **EPHA7** | **0.55** |  | **ERK5** | **nb** |  | **FLT3** | **0.1** |
| **EPHA8** | **0** |  | **ERK8** | **nb** |  | **FLT3(D835H)** | **0.1** |
| **EPHB1** | **0.5** |  | **ERN1** | **1.4** |  | **FLT3(D835Y)** | **0.85** |
| **EPHB2** | **0.1** |  | **FAK** | **nb** |  | **FLT3(ITD)** | **0.1** |
| **EPHB3** | **0.25** |  | **FER** | **5.6** |  | **FLT3(K663Q)** | **0** |
| **EPHB4** | **1.6** |  | **FES** | **0.8** |  | **FLT3(N841I)** | **0.15** |
| **EPHB6** | **8.8** |  | **FGFR1** | **2.2** |  | **FLT4** | **0.15** |
| **ERBB2** | **0.5** |  | **FGFR2** | **4.3** |  | **FRK** | **0.55** |
| **ERBB3** | **nb** |  | **FGFR3** | **nb** |  | **FYN** | **2.2** |
| **ERBB4** | **1.8** |  | **FGFR3(G697C)** | **nb** |  | **GAK** | **4.3** |
| **ERK1** | **nb** |  | **FGFR4** | **8.7** |  | **GCN2(Kin.Dom.2.S808G)** | **0.95** |
| **ERK2** | **nb** |  | **FGR** | **0.2** |  | **GSK3A** | **nb** |
| **ERK3** | **nb** |  | **FLT1** | **1** |  | **GSK3B** | **nb** |
| **ERK4** | **nb** |  | **FLT3** | **0.65** |  | **HCK** | **0.3** |
| **ERK5** | **nb** |  | **FLT3(D835H)** | **1.4** |  | **HIPK1** | **nb** |
| **ERK8** | **1.8** |  | **FLT3(D835Y)** | **3.3** |  | **IGF1R** | **nb** |
| **ERN1** | **nb** |  | **FLT3(ITD)** | **0.4** |  | **IKK-alpha** | **0.75** |
| **FAK** | **nb** |  | **FLT3(K663Q)** | **1** |  | **IKK-beta** | **5.9** |
| **FER** | **11** |  | **FLT3(N841I)** | **0.75** |  | **IKK-epsilon** | **nb** |
| **FES** | **0.75** |  | **FLT4** | **0** |  | **INSR** | **nb** |
| **FGFR1** | **0.25** |  | **FRK** | **0.2** |  | **INSRR** | **nb** |
| **FGFR2** | **0.5** |  | **FYN** | **0.25** |  | **IRAK3** | **nb** |
| **FGFR3** | **0.3** |  | **GAK** | **1.6** |  | **ITK** | **21** |
| **FGFR3(G697C)** | **0.05** |  | **GCN2(Kin.Dom.2.S808G)** | **0.3** |  | **JAK1(Kin.Dom.1)** | **nb** |
| **FGFR4** | **0** |  | **GRK1** | **nb** |  | **JAK1(Kin.Dom.2)** | **11** |
| **FGR** | **0** |  | **GRK4** | **nb** |  | **JAK2(Kin.Dom.2)** | **12** |
| **FLT1** | **0.5** |  | **GRK7** | **16** |  | **JAK3(Kin.Dom.2)** | **3.5** |
| **FLT3** | **0.25** |  | **GSK3A** | **nb** |  | **JNK1** | **6.6** |
| **FLT3(D835H)** | **0.15** |  | **GSK3B** | **nb** |  | **JNK2** | **1.1** |
| **FLT3(D835Y)** | **0.5** |  | **HCK** | **0.2** |  | **JNK3** | **nb** |
| **FLT3(ITD)** | **0.1** |  | **HIPK1** | **nb** |  | **KIT** | **0** |
| **FLT3(K663Q)** | **0** |  | **HIPK2** | **nb** |  | **KIT(D816V)** | **0.3** |
| **FLT3(N841I)** | **0** |  | **HIPK3** | **nb** |  | **KIT(V559D)** | **0** |
| **FLT3(R834Q)** | **4.2** |  | **HIPK4** | **25** |  | **KIT(V559D.T670I)** | **0** |
| **FLT4** | **0** |  | **HPK1** | **0.75** |  | **KIT(V559D.V654A)** | **0.9** |
| **FRK** | **0.15** |  | **HUNK** | **nb** |  | **LATS1** | **nb** |
| **FYN** | **0.35** |  | **ICK** | **nb** |  | **LATS2** | **nb** |
| **GAK** | **7.4** |  | **IGF1R** | **nb** |  | **LCK** | **0.1** |
| **GCN2(Kin.Dom.2.S808G)** | **1.3** |  | **IKK-alpha** | **3.3** |  | **LIMK1** | **4.1** |
| **GRK1** | **nb** |  | **IKK-beta** | **1.2** |  | **LIMK2** | **6.6** |
| **GRK4** | **nb** |  | **IKK-epsilon** | **nb** |  | **LKB1** | **29** |
| **GRK7** | **nb** |  | **INSR** | **12** |  | **LOK** | **0** |
| **GSK3A** | **nb** |  | **INSRR** | **8.8** |  | **LTK** | **nb** |
| **GSK3B** | **nb** |  | **IRAK1** | **17** |  | **LYN** | **0.25** |
| **HCK** | **0.05** |  | **IRAK3** | **nb** |  | **MAP3K3** | **4** |
| **HIPK1** | **nb** |  | **ITK** | **8.6** |  | **MAP3K4** | **nb** |
| **HIPK2** | **nb** |  | **JAK1(JH1domain-catalytic)** | **14** |  | **MAP3K5** | **nb** |
| **HIPK3** | **18** |  | **JAK1(JH2domain-pseudokinase)** | **nb** |  | **MAP4K1** | **0.1** |
| **HIPK4** | **2.2** |  | **JAK2(JH1domain-catalytic)** | **11** |  | **MAP4K2** | **0.25** |
| **HPK1** | **0.4** |  | **JAK3(JH1domain-catalytic)** | **3.7** |  | **MAP4K3** | **4.2** |
| **HUNK** | **nb** |  | **JNK1** | **nb** |  | **MAP4K4** | **0.3** |
| **ICK** | **nb** |  | **JNK2** | **0.25** |  | **MAP4K5** | **1.8** |
| **IGF1R** | **nb** |  | **JNK3** | **32** |  | **MAPKAPK2** | **nb** |
| **IKK-alpha** | **0** |  | **KIT** | **0** |  | **MAPKAPK5** | **nb** |
| **IKK-beta** | **0** |  | **KIT(D816V)** | **0.75** |  | **MARK1** | **nb** |
| **IKK-epsilon** | **nb** |  | **KIT(L576P)** | **0** |  | **MARK2** | **nb** |
| **INSR** | **nb** |  | **KIT(V559D)** | **0.1** |  | **MARK3** | **29** |
| **INSRR** | **nb** |  | **KIT(V559D.T670I)** | **0.05** |  | **MARK4** | **25** |
| **IRAK1** | **1.2** |  | **KIT(V559D.V654A)** | **5.4** |  | **MEK1** | **nb** |
| **IRAK3** | **nb** |  | **LATS1** | **nb** |  | **MEK2** | **nb** |
| **IRAK4** | **nb** |  | **LATS2** | **nb** |  | **MEK3** | **nb** |
| **ITK** | **nb** |  | **LCK** | **0.2** |  | **MEK4** | **nb** |
| **JAK1(JH1domain-catalytic)** | **6.2** |  | **LIMK1** | **23** |  | **MEK6** | **nb** |
| **JAK1(JH2domain-pseudokinase)** | **nb** |  | **LIMK2** | **31** |  | **MELK** | **22** |
| **JAK2(JH1domain-catalytic)** | **3** |  | **LKB1** | **nb** |  | **MERTK** | **nb** |
| **JAK3(JH1domain-catalytic)** | **2.3** |  | **LOK** | **0.05** |  | **MET** | **nb** |
| **JNK1** | **0.55** |  | **LTK** | **nb** |  | **MINK** | **4.2** |
| **JNK2** | **0** |  | **LYN** | **0.1** |  | **MKNK1** | **nb** |
| **JNK3** | **0.95** |  | **LZK** | **15** |  | **MKNK2** | **0.75** |
| **KIT** | **0** |  | **MAK** | **nb** |  | **MLCK** | **5.9** |
| **KIT(A829P)** | **27** |  | **MAP3K1** | **nb** |  | **MLK1** | **0.6** |
| **KIT(D816H)** | **6** |  | **MAP3K15** | **nb** |  | **MLK2** | **20** |
| **KIT(D816V)** | **2** |  | **MAP3K2** | **3.8** |  | **MLK3** | **0.8** |
| **KIT(L576P)** | **0** |  | **MAP3K3** | **0.25** |  | **MRCKA** | **12** |
| **KIT(V559D)** | **0** |  | **MAP3K4** | **nb** |  | **MRCKB** | **30** |
| **KIT(V559D.T670I)** | **0** |  | **MAP4K2** | **0.6** |  | **MST1** | **26** |
| **KIT(V559D.V654A)** | **4.2** |  | **MAP4K3** | **20** |  | **MST1R** | **nb** |
| **LATS1** | **20** |  | **MAP4K4** | **6.4** |  | **MST2** | **10** |
| **LATS2** | **7.6** |  | **MAP4K5** | **0.75** |  | **MST3** | **nb** |
| **LCK** | **0.05** |  | **MAPKAPK2** | **nb** |  | **MST4** | **32** |
| **LIMK1** | **3** |  | **MAPKAPK5** | **nb** |  | **MUSK** | **0.05** |
| **LIMK2** | **11** |  | **MARK1** | **nb** |  | **MYLK** | **nb** |
| **LKB1** | **nb** |  | **MARK2** | **nb** |  | **MYLK2** | **0.25** |
| **LOK** | **0** |  | **MARK3** | **nb** |  | **MYO3A** | **nb** |
| **LRRK2** | **3.4** |  | **MARK4** | **nb** |  | **MYO3B** | **30** |
| **LRRK2(G2019S)** | **7.5** |  | **MAST1** | **nb** |  | **NDR2** | **nb** |
| **LTK** | **4.5** |  | **MEK1** | **nb** |  | **NEK1** | **nb** |
| **LYN** | **0.05** |  | **MEK2** | **nb** |  | **NEK2** | **nb** |
| **LZK** | **7.4** |  | **MEK3** | **nb** |  | **NEK5** | **7** |
| **MAK** | **9** |  | **MEK4** | **nb** |  | **NEK6** | **nb** |
| **MAP3K1** | **nb** |  | **MEK6** | **nb** |  | **NEK7** | **nb** |
| **MAP3K15** | **nb** |  | **MELK** | **nb** |  | **NEK9** | **28** |
| **MAP3K2** | **0.15** |  | **MERTK** | **nb** |  | **NLK** | **6.8** |
| **MAP3K3** | **1.4** |  | **MET** | **nb** |  | **p38-alpha** | **0.15** |
| **MAP3K4** | **nb** |  | **MET(M1250T)** | **nb** |  | **p38-beta** | **0** |
| **MAP4K2** | **0** |  | **MET(Y1235D)** | **nb** |  | **p38-delta** | **nb** |
| **MAP4K3** | **2.6** |  | **MINK** | **0.65** |  | **p38-gamma** | **13** |
| **MAP4K4** | **3.3** |  | **MKNK1** | **14** |  | **PAK1** | **nb** |
| **MAP4K5** | **3** |  | **MKNK2** | **nb** |  | **PAK2** | **nb** |
| **MAPKAPK2** | **nb** |  | **MLCK** | **nb** |  | **PAK3** | **30** |
| **MAPKAPK5** | **nb** |  | **MLK1** | **nb** |  | **PAK4** | **nb** |
| **MARK1** | **nb** |  | **MLK2** | **nb** |  | **PAK6** | **nb** |
| **MARK2** | **nb** |  | **MLK3** | **nb** |  | **PAK7/PAK5** | **nb** |
| **MARK3** | **nb** |  | **MRCKA** | **nb** |  | **PCTK1** | **nb** |
| **MARK4** | **nb** |  | **MRCKB** | **nb** |  | **PCTK2** | **nb** |
| **MAST1** | **nb** |  | **MST1** | **21** |  | **PCTK3** | **nb** |
| **MEK1** | **nb** |  | **MST1R** | **nb** |  | **PDGFRA** | **0** |
| **MEK2** | **nb** |  | **MST2** | **nb** |  | **PDGFRB** | **0** |
| **MEK3** | **nb** |  | **MST3** | **nb** |  | **PDPK1** | **nb** |
| **MEK4** | **nb** |  | **MST4** | **12** |  | **PFTAIRE2** | **nb** |
| **MEK5** | **0.4** |  | **MUSK** | **0.1** |  | **PFTK1** | **nb** |
| **MEK6** | **nb** |  | **MYLK** | **nb** |  | **PHKG1** | **nb** |
| **MELK** | **8.1** |  | **MYLK2** | **nb** |  | **PHKG2** | **nb** |
| **MERTK** | **5.2** |  | **MYO3A** | **17** |  | **PIK3C2B** | **nb** |
| **MET** | **nb** |  | **MYO3B** | **nb** |  | **PIK3CA** | **nb** |
| **MET(M1250T)** | **nb** |  | **NDR1** | **nb** |  | **PIK3CA(E545K)** | **nb** |
| **MET(Y1235D)** | **nb** |  | **NDR2** | **11** |  | **PIK3CB** | **nb** |
| **MINK** | **2.5** |  | **NEK1** | **nb** |  | **PIK3CD** | **nb** |
| **MKK7** | **11** |  | **NEK2** | **nb** |  | **PIK3CG** | **nb** |
| **MKNK1** | **0.25** |  | **NEK5** | **nb** |  | **PIM1** | **nb** |
| **MKNK2** | **0** |  | **NEK6** | **nb** |  | **PIM2** | **nb** |
| **MLCK** | **nb** |  | **NEK7** | **nb** |  | **PIM3** | **nb** |
| **MLK1** | **nb** |  | **NEK9** | **nb** |  | **PIP5K1A** | **15** |
| **MLK2** | **nb** |  | **NIM1** | **nb** |  | **PIP5K2B** | **5** |
| **MLK3** | **17** |  | **NLK** | **7.2** |  | **PKAC-alpha** | **nb** |
| **MRCKA** | **nb** |  | **OSR1** | **nb** |  | **PKAC-beta** | **nb** |
| **MRCKB** | **nb** |  | **p38-alpha** | **0** |  | **PKMYT1** | **nb** |
| **MST1** | **23** |  | **p38-beta** | **0** |  | **PKN1** | **nb** |
| **MST1R** | **nb** |  | **p38-delta** | **29** |  | **PKN2** | **23** |
| **MST2** | **nb** |  | **p38-gamma** | **3.6** |  | **PLK1** | **nb** |
| **MST3** | **2.8** |  | **PAK1** | **nb** |  | **PLK3** | **nb** |
| **MST4** | **21** |  | **PAK2** | **nb** |  | **PLK4** | **nb** |
| **MTOR** | **nb** |  | **PAK3** | **nb** |  | **PRKCD** | **nb** |
| **MUSK** | **0.05** |  | **PAK4** | **nb** |  | **PRKCE** | **nb** |
| **MYLK** | **nb** |  | **PAK6** | **nb** |  | **PRKCH** | **nb** |
| **MYLK2** | **3.4** |  | **PAK7** | **nb** |  | **PRKCQ** | **nb** |
| **MYLK4** | **nb** |  | **PCTK1** | **nb** |  | **PRKD1** | **nb** |
| **MYO3A** | **3.2** |  | **PCTK2** | **nb** |  | **PRKD2** | **32** |
| **MYO3B** | **0** |  | **PCTK3** | **nb** |  | **PRKD3** | **nb** |
| **NDR1** | **4** |  | **PDGFRA** | **0** |  | **PRKG1** | **nb** |
| **NDR2** | **8.7** |  | **PDGFRB** | **0** |  | **PRKG2** | **nb** |
| **NEK1** | **nb** |  | **PDPK1** | **nb** |  | **PRKR** | **nb** |
| **NEK11** | **9.1** |  | **PFTAIRE2** | **nb** |  | **PRKX** | **nb** |
| **NEK2** | **nb** |  | **PFTK1** | **nb** |  | **PTK2** | **nb** |
| **NEK3** | **53** |  | **PHKG1** | **nb** |  | **PTK2B** | **5.5** |
| **NEK4** | **6.4** |  | **PHKG2** | **nb** |  | **PTK6** | **17** |
| **NEK5** | **4.8** |  | **PIK3C2B** | **nb** |  | **RAF1** | **2.4** |
| **NEK6** | **90** |  | **PIK3C2G** | **nb** |  | **RET** | **0** |
| **NEK7** | **88** |  | **PIK3CA** | **nb** |  | **RET(M918T)** | **0** |
| **NEK9** | **41** |  | **PIK3CA(C420R)** | **nb** |  | **RET(V804L)** | **0** |
| **NIM1** | **97** |  | **PIK3CA(E542K)** | **nb** |  | **RET(V804M)** | **0** |
| **NLK** | **8.2** |  | **PIK3CA(E545A)** | **nb** |  | **RIOK1** | **0.2** |
| **OSR1** | **48** |  | **PIK3CA(E545K)** | **nb** |  | **RIOK2** | **33** |
| **p38-alpha** | **0** |  | **PIK3CA(H1047L)** | **nb** |  | **RIOK3** | **0.35** |
| **p38-beta** | **0** |  | **PIK3CA(H1047Y)** | **nb** |  | **RIPK1** | **0.05** |
| **p38-delta** | **2.5** |  | **PIK3CA(M1043I)** | **nb** |  | **RIPK2** | **1.6** |
| **p38-gamma** | **2.7** |  | **PIK3CA(Q546K)** | **nb** |  | **RIPK4** | **nb** |
| **PAK1** | **81** |  | **PIK3CB** | **nb** |  | **ROCK2** | **nb** |
| **PAK2** | **84** |  | **PIK3CD** | **nb** |  | **ROS1** | **nb** |
| **PAK3** | **3.3** |  | **PIK3CG** | **nb** |  | **RPS6KA1(Kin.Dom.1)** | **nb** |
| **PAK4** | **100** |  | **PIK4CB** | **nb** |  | **RPS6KA1(Kin.Dom.2)** | **nb** |
| **PAK6** | **100** |  | **PIM1** | **nb** |  | **RPS6KA2(Kin.Dom.1)** | **7.4** |
| **PAK7** | **83** |  | **PIM2** | **22** |  | **RPS6KA2(Kin.Dom.2)** | **nb** |
| **PCTK1** | **8.4** |  | **PIM3** | **nb** |  | **RPS6KA3(Kin.Dom.1)** | **nb** |
| **PCTK2** | **8.2** |  | **PIP5K1A** | **nb** |  | **RPS6KA4(Kin.Dom.1)** | **nb** |
| **PCTK3** | **6.2** |  | **PIP5K2B** | **nb** |  | **RPS6KA4(Kin.Dom.2)** | **nb** |
| **PDGFRA** | **0** |  | **PKAC-alpha** | **7.4** |  | **RPS6KA5(Kin.Dom.1)** | **nb** |
| **PDGFRB** | **0** |  | **PKAC-beta** | **8** |  | **RPS6KA5(Kin.Dom.2)** | **nb** |
| **PDPK1** | **84** |  | **PKMYT1** | **nb** |  | **RPS6KA6(Kin.Dom.1)** | **nb** |
| **PFCDPK1(P.falciparum)** | **0.05** |  | **PKN1** | **nb** |  | **RPS6KA6(Kin.Dom.2)** | **nb** |
| **PFPK5(P.falciparum)** | **89** |  | **PKN2** | **nb** |  | **SgK085** | **8.5** |
| **PFTAIRE2** | **6.4** |  | **PLK1** | **nb** |  | **SgK110** | **29** |
| **PFTK1** | **0.85** |  | **PLK2** | **nb** |  | **SLK** | **0.85** |
| **PHKG1** | **88** |  | **PLK3** | **nb** |  | **SNARK** | **nb** |
| **PHKG2** | **92** |  | **PLK4** | **nb** |  | **SNF1LK** | **0.3** |
| **PIK3C2B** | **100** |  | **PRKCD** | **1.2** |  | **SNF1LK2** | **6.4** |
| **PIK3C2G** | **100** |  | **PRKCE** | **5** |  | **SRC** | **0.25** |
| **PIK3CA** | **100** |  | **PRKCH** | **4.6** |  | **SRMS** | **23** |
| **PIK3CA(C420R)** | **72** |  | **PRKCQ** | **22** |  | **SRPK1** | **0.25** |
| **PIK3CA(E542K)** | **100** |  | **PRKD1** | **nb** |  | **SRPK2** | **28** |
| **PIK3CA(E545A)** | **93** |  | **PRKD2** | **nb** |  | **SRPK3** | **22** |
| **PIK3CA(E545K)** | **85** |  | **PRKD3** | **nb** |  | **STK16** | **18** |
| **PIK3CA(H1047L)** | **100** |  | **PRKG1** | **nb** |  | **STK33** | **23** |
| **PIK3CA(H1047Y)** | **81** |  | **PRKG2** | **nb** |  | **STK35** | **nb** |
| **PIK3CA(I800L)** | **72** |  | **PRKR** | **nb** |  | **STK36** | **0.05** |
| **PIK3CA(M1043I)** | **83** |  | **PRKX** | **nb** |  | **SYK** | **1.8** |
| **PIK3CA(Q546K)** | **100** |  | **PRP4** | **nb** |  | **TAK1** | **0.1** |
| **PIK3CB** | **100** |  | **PYK2** | **2.4** |  | **TAOK1** | **6.5** |
| **PIK3CD** | **76** |  | **QSK** | **nb** |  | **TAOK3** | **0.1** |
| **PIK3CG** | **100** |  | **RAF1** | **1.4** |  | **TEC** | **1.2** |
| **PIK4CB** | **100** |  | **RET** | **0.05** |  | **TESK1** | **4.2** |
| **PIM1** | **84** |  | **RET(M918T)** | **0** |  | **TGFBR1** | **nb** |
| **PIM2** | **100** |  | **RET(V804L)** | **0** |  | **TGFBR2** | **2.8** |
| **PIM3** | **100** |  | **RET(V804M)** | **0** |  | **TIE1** | **1.2** |
| **PIP5K1A** | **79** |  | **RIOK1** | **nb** |  | **TIE2** | **0.1** |
| **PIP5K1C** | **90** |  | **RIOK2** | **nb** |  | **TLK1** | **nb** |
| **PIP5K2B** | **58** |  | **RIOK3** | **nb** |  | **TLK2** | **nb** |
| **PIP5K2C** | **100** |  | **RIPK1** | **0.45** |  | **TNIK** | **13** |
| **PKAC-alpha** | **8.4** |  | **RIPK2** | **3.1** |  | **TNK1** | **0.8** |
| **PKAC-beta** | **4.1** |  | **RIPK4** | **nb** |  | **TNK2** | **0.5** |
| **PKMYT1** | **85** |  | **ROCK1** | **0** |  | **TNNI3K** | **0.9** |
| **PKN1** | **85** |  | **ROCK2** | **18** |  | **TRKA** | **8.2** |
| **PKN2** | **36** |  | **ROS1** | **nb** |  | **TRKB** | **1.6** |
| **PKNB(M.tuberculosis)** | **87** |  | **RPS6KA1(Kin.Dom.1-N-terminal)** | **nb** |  | **TRKC** | **5.3** |
| **PLK1** | **58** |  | **RPS6KA1(Kin.Dom.2-C-terminal)** | **nb** |  | **TSSK1** | **nb** |
| **PLK2** | **100** |  | **RPS6KA2(Kin.Dom.1-N-terminal)** | **nb** |  | **TTK** | **nb** |
| **PLK3** | **90** |  | **RPS6KA2(Kin.Dom.2-C-terminal)** | **nb** |  | **TXK** | **0.9** |
| **PLK4** | **88** |  | **RPS6KA3(Kin.Dom.1-N-terminal)** | **nb** |  | **TYK2(Kin.Dom.1)** | **nb** |
| **PRKCD** | **39** |  | **RPS6KA4(Kin.Dom.1-N-terminal)** | **6.3** |  | **TYK2(Kin.Dom.2)** | **7** |
| **PRKCE** | **54** |  | **RPS6KA4(Kin.Dom.2-C-terminal)** | **nb** |  | **TYRO3** | **nb** |
| **PRKCH** | **67** |  | **RPS6KA5(Kin.Dom.1-N-terminal)** | **11** |  | **ULK1** | **8.7** |
| **PRKCI** | **42** |  | **RPS6KA5(Kin.Dom.2-C-terminal)** | **nb** |  | **ULK2** | **27** |
| **PRKCQ** | **55** |  | **RPS6KA6(Kin.Dom.1-N-terminal)** | **nb** |  | **ULK3** | **23** |
| **PRKD1** | **51** |  | **RPS6KA6(Kin.Dom.2-C-terminal)** | **nb** |  | **VEGFR2** | **0.35** |
| **PRKD2** | **56** |  | **SBK1** | **nb** |  | **WEE1** | **nb** |
| **PRKD3** | **22** |  | **SgK085** | **nb** |  | **WEE2** | **15** |
| **PRKG1** | **100** |  | **SgK110** | **nb** |  | **YANK2** | **nb** |
| **PRKG2** | **97** |  | **SIK** | **0.2** |  | **YANK3** | **nb** |
| **PRKR** | **66** |  | **SIK2** | **13** |  | **YES** | **0.85** |
| **PRKX** | **3.6** |  | **SLK** | **5** |  | **YSK1** | **nb** |
| **PRP4** | **54** |  | **SNARK** | **nb** |  | **ZAK** | **0.35** |
| **PYK2** | **0.4** |  | **SRC** | **0.1** |  | **ZAP70** | **nb** |
| **QSK** | **70** |  | **SRMS** | **2.8** |  |  |  |
| **RAF1** | **0.5** |  | **SRPK1** | **nb** |  |  |  |
| **RET** | **0** |  | **SRPK2** | **nb** |  |  |  |
| **RET(M918T)** | **0** |  | **SRPK3** | **nb** |  |  |  |
| **RET(V804L)** | **0** |  | **STK16** | **nb** |  |  |  |
| **RET(V804M)** | **0** |  | **STK33** | **nb** |  |  |  |
| **RIOK1** | **90** |  | **STK35** | **21** |  |  |  |
| **RIOK2** | **0.5** |  | **STK36** | **0.2** |  |  |  |
| **RIOK3** | **70** |  | **STK39** | **nb** |  |  |  |
| **RIPK1** | **0.05** |  | **SYK** | **4.2** |  |  |  |
| **RIPK2** | **0.45** |  | **TAK1** | **0** |  |  |  |
| **RIPK4** | **48** |  | **TAO1** | **12** |  |  |  |
| **RIPK5** | **27** |  | **TAOK1** | **nb** |  |  |  |
| **ROCK1** | **43** |  | **TAOK3** | **2.8** |  |  |  |
| **ROCK2** | **19** |  | **TBK1** | **nb** |  |  |  |
| **ROS1** | **46** |  | **TEC** | **1.7** |  |  |  |
| **RPS6KA4(Kin.Dom.1-N-terminal)** | **30** |  | **TESK1** | **nb** |  |  |  |
| **RPS6KA4(Kin.Dom.2-C-terminal)** | **99** |  | **TGFBR1** | **nb** |  |  |  |
| **RPS6KA5(Kin.Dom.1-N-terminal)** | **59** |  | **TGFBR2** | **4.6** |  |  |  |
| **RPS6KA5(Kin.Dom.2-C-terminal)** | **100** |  | **TIE1** | **1.4** |  |  |  |
| **RSK1(Kin.Dom.1-N-terminal)** | **53** |  | **TIE2** | **0.45** |  |  |  |
| **RSK1(Kin.Dom.2-C-terminal)** | **7.2** |  | **TLK1** | **nb** |  |  |  |
| **RSK2(Kin.Dom.1-N-terminal)** | **16** |  | **TLK2** | **nb** |  |  |  |
| **RSK3(Kin.Dom.1-N-terminal)** | **9.5** |  | **TNIK** | **11** |  |  |  |
| **RSK3(Kin.Dom.2-C-terminal)** | **30** |  | **TNK1** | **7.7** |  |  |  |
| **RSK4(Kin.Dom.1-N-terminal)** | **47** |  | **TNK2** | **2.1** |  |  |  |
| **RSK4(Kin.Dom.2-C-terminal)** | **6.8** |  | **TNNI3K** | **3.8** |  |  |  |
| **S6K1** | **11** |  | **TRKA** | **4.4** |  |  |  |
| **SBK1** | **88** |  | **TRKB** | **4** |  |  |  |
| **SgK110** | **100** |  | **TRKC** | **0.85** |  |  |  |
| **SGK3** | **6.2** |  | **TSSK1B** | **nb** |  |  |  |
| **SIK** | **0.45** |  | **TTK** | **nb** |  |  |  |
| **SIK2** | **21** |  | **TXK** | **1.8** |  |  |  |
| **SLK** | **0.1** |  | **TYK2(JH1domain-catalytic)** | **9.8** |  |  |  |
| **SNARK** | **99** |  | **TYK2(JH2domain-pseudokinase)** | **nb** |  |  |  |
| **SNRK** | **34** |  | **TYRO3** | **nb** |  |  |  |
| **SRC** | **0** |  | **ULK1** | **nb** |  |  |  |
| **SRMS** | **0** |  | **ULK2** | **nb** |  |  |  |
| **SRPK1** | **100** |  | **ULK3** | **0.95** |  |  |  |
| **SRPK2** | **81** |  | **VEGFR2** | **1.8** |  |  |  |
| **SRPK3** | **45** |  | **WEE1** | **nb** |  |  |  |
| **STK16** | **100** |  | **WEE2** | **nb** |  |  |  |
| **STK33** | **14** |  | **YANK2** | **nb** |  |  |  |
| **STK35** | **1.6** |  | **YANK3** | **nb** |  |  |  |
| **STK36** | **0.4** |  | **YES** | **0.1** |  |  |  |
| **STK39** | **89** |  | **YSK1** | **nb** |  |  |  |
| **SYK** | **13** |  | **YSK4** | **21** |  |  |  |
| **TAK1** | **0.2** |  | **ZAK** | **3** |  |  |  |
| **TAOK1** | **0.25** |  | **ZAP70** | **nb** |  |  |  |
| **TAOK2** | **0.15** |  |  |  |  |  |  |
| **TAOK3** | **0** |  |  |  |  |  |  |
| **TBK1** | **100** |  |  |  |  |  |  |
| **TEC** | **3.7** |  |  |  |  |  |  |
| **TESK1** | **60** |  |  |  |  |  |  |
| **TGFBR1** | **100** |  |  |  |  |  |  |
| **TGFBR2** | **1.3** |  |  |  |  |  |  |
| **TIE1** | **1.6** |  |  |  |  |  |  |
| **TIE2** | **0.05** |  |  |  |  |  |  |
| **TLK1** | **93** |  |  |  |  |  |  |
| **TLK2** | **100** |  |  |  |  |  |  |
| **TNIK** | **1.8** |  |  |  |  |  |  |
| **TNK1** | **0** |  |  |  |  |  |  |
| **TNK2** | **11** |  |  |  |  |  |  |
| **TNNI3K** | **0** |  |  |  |  |  |  |
| **TRKA** | **0.35** |  |  |  |  |  |  |
| **TRKB** | **1.6** |  |  |  |  |  |  |
| **TRKC** | **0.35** |  |  |  |  |  |  |
| **TRPM6** | **49** |  |  |  |  |  |  |
| **TSSK1B** | **92** |  |  |  |  |  |  |
| **TTK** | **16** |  |  |  |  |  |  |
| **TXK** | **0.3** |  |  |  |  |  |  |
| **TYK2(JH1domain-catalytic)** | **4.2** |  |  |  |  |  |  |
| **TYK2(JH2domain-pseudokinase)** | **99** |  |  |  |  |  |  |
| **TYRO3** | **100** |  |  |  |  |  |  |
| **ULK1** | **24** |  |  |  |  |  |  |
| **ULK2** | **50** |  |  |  |  |  |  |
| **ULK3** | **0.25** |  |  |  |  |  |  |
| **VEGFR2** | **0.25** |  |  |  |  |  |  |
| **VRK2** | **95** |  |  |  |  |  |  |
| **WEE1** | **100** |  |  |  |  |  |  |
| **WEE2** | **100** |  |  |  |  |  |  |
| **YANK1** | **81** |  |  |  |  |  |  |
| **YANK2** | **100** |  |  |  |  |  |  |
| **YANK3** | **75** |  |  |  |  |  |  |
| **YES** | **0.1** |  |  |  |  |  |  |
| **YSK1** | **16** |  |  |  |  |  |  |
| **YSK4** | **0.1** |  |  |  |  |  |  |
| **ZAK** | **0.4** |  |  |  |  |  |  |
| **ZAP70** | **62** |  |  |  |  |  |  |
